# The progress on stevia (*Stevia rebaudiana* Bertoni): chemical composition, pharmacokinetics, pharmacological effects, safety, applications, and biosynthesis

**DOI:** 10.3389/fnut.2026.1728578

**Published:** 2026-02-16

**Authors:** Zhi Peng, Shuang Zhan, Xiulian Yang, Sirui Huang, Huaxue Huang, Wei Wang

**Affiliations:** 1 Hunan Huacheng Biological Resources Co., Ltd., Changsha, Hunan, China; 2 Hunan Natural Sweetener Engineering Technology Research Center, Changsha, Hunan, China; 3 School of Pharmacy, Macau University of Science and Technology, Macau, China; 4 TCM and Ethnomedicine Innovation and Development International Laboratory, School of Pharmacy, Innovative Materia Medica Research Institute, Hunan University of Chinese Medicine, Changsha, Hunan, China; 5 Modernization Industry College for Innovative Chinese Medicine, Hunan University of Chinese Medicine, Changsha, Hunan, China

**Keywords:** stevia, steviol glycosides, stevioside, rebaudioside, pharmacological properties, safety, biological synthesis

## Abstract

**Background:**

*Stevia rebaudiana* Bertoni (stevia) is renowned for its natural sweetening properties, attributed to a complex phytochemical profile rich in steviol glycosides (SGs), flavonoids, and phenolic compounds.

**Methods:**

A systematic search of PubMed, Web of Science, and CNKI (Jan 2005–Sep 2025) using keywords related to Stevia yielded over 17,600 records. After deduplication and screening, around 200 full-text articles were evaluated. Additional key references were identified through citation tracking. Ultimately, over 140 sources were gathered to create a comprehensive overview prioritizing significant evidence.

**Results:**

The findings reveal that SGs are not metabolized in the upper gastrointestinal tract but are hydrolyzed exclusively by gut microbiota to steviol, accounting for their negligible caloric impact. Stevia extracts and their constituents exhibit a broad spectrum of potent pharmacological activities, including antioxidant, anti-inflammatory, anti-hyperglycemic, anti-hyperlipidemic, and hepatorenal protective effects, mediated through diverse molecular mechanisms. Toxicological evaluations confirm the safety of SGs, establishing an Acceptable Daily Intake of 4 mg kg^–1^ body weight. Beyond sweetening, Stevia finds applications in functional foods, animal feed, and agriculture. Furthermore, advanced biosynthetic strategies are being developed to overcome the sensory limitations of traditional SGs, with a focus on the enzymatic production of next-generation glycosides like Rebaudioside M.

**Conclusion:**

This review integrates multidisciplinary evidence on SGs—encompassing chemistry, pharmacology, toxicology, applications, biosynthesis, and regulation—to create a cohesive synthesis and actionable visual frameworks. These resources aim to offer an evidence-based foundation for industry professionals, regulatory bodies, and researchers to effectively address critical priorities and identified gaps.

## 1 Introduction

The global rise in obesity, type 2 diabetes mellitus (T2DM), and other metabolic syndromes is inextricably linked to the excessive consumption of refined sugars, posing an urgent public health challenge worldwide. This has catalyzed a fervent search for sugar substitutes within the food industry. Although artificial sweeteners like aspartame and sucralose dominate the market due to their intense sweetness and low cost (1), growing epidemiological evidence raises concerns about their potential to disrupt gut microbiota and impair glucose homeostasis, casting doubt on their long-term safety (2, 3). This rapidly expanding concern has sparked increased interest in natural, non-nutritive sweeteners, which are considered safer and sustainable alternatives.

*Stevia rebaudiana* Bertoni is a natural, zero-calorie sweetener derived from its leaves, which are the primary source of sweet-tasting steviol glycosides (SGs). The leaves contain a complex profile of diterpene glycosides, primarily stevioside (STE) and various rebaudiosides (e.g., Reb A, Reb D, Reb M), which are all derivatives of a common steviol aglycone backbone. Beyond steviol glycosides (SGs), stevia leaves are a rich source of bioactive secondary metabolites, including flavonoids, phenolic acids like chlorogenic acid, and essential oils, which contribute to its potent antioxidant capacity (4–6).

A critical aspect of stevia’s functionality is its distinct pharmacokinetic profile. SGs are not hydrolyzed by human digestive enzymes and pass intact through the upper gastrointestinal tract. Upon reaching the colon, they are extensively metabolized by the gut microbiota into steviol, which is subsequently absorbed, conjugated in the liver to form steviol glucuronide, and excreted primarily in urine (7–9). This microbial-dependent metabolism is the fundamental basis for its “zero-calorie” claim, as the sugars released are fermented by colonic bacteria into short-chain fatty acids that provide minimal net energy to the host.

Extensive research has revealed a broad spectrum of pharmacological activities associated with stevia and its phytochemicals. These include anti-hyperglycemic effects through mechanisms such as enhanced insulin secretion and modulation of glucose transporters (10, 11), anti-hyperlipidemic properties (12), and antihypertensive actions linked to vasodilation and angiotensin-converting enzyme (ACE) inhibition (13, 14). Furthermore, stevia exhibits anti-inflammatory (15, 16), antioxidant (17), anti-caries (18, 19), anti-tumor (20), and anti-diarrheal effects (21, 22). Toxicological studies have consistently supported its safety, establishing an acceptable daily intake (ADI) of 4 mg⋅kg^–1^ body weight and showing no evidence of mutagenicity or carcinogenicity (23–25).

Despite its promising health benefits, substantial challenges and research gaps persist. The bitter aftertaste inherent to major SGs, such as STE, restricts consumer acceptance, driving the search for more palatable alternatives like Reb M. However, this compound is scarce in the plant and necessitates efficient biosynthetic approaches (26). Although safety evaluations generally support its use within acceptable daily intake limits, subtle variations remain, and further investigation is required to clarify long-term effects, particularly on gut microbiota and vulnerable populations (27). Critically, the translation from *in vitro* and animal studies to robust human clinical trials is still incomplete, and the synergistic interactions between complex mixtures in whole leaf extracts and purified SGs remain poorly understood.

The uniqueness of this review lies in its extensive coverage and its systematic and critical synthesis of different disciplines, aiming to fill the gaps in the existing literature. It is not merely a descriptive summary; rather, it incorporates a methodological approach through original comparative tables and diagrams that distill complex information regarding chemical properties, Pharmacological and toxicological experiments, and production pathways. Importantly, this review adopts a translational perspective, rigorously assessing the quality of evidence, clearly addressing contradictions and focal points within the research, and summarizing actionable research gaps along with their implications for the industry and regulatory bodies in a clear action plan. This integrative approach offers a strategic analysis that connects basic science, advanced biotechnology, and practical applications, suggesting a contribution that may extend beyond existing specialized reviews.

## 2 Methods

This review is a comprehensive narrative synthesis of the current scientific literature on *S. rebaudiana*. Its primary aim is to integrate and critically analyze evidence spanning chemistry, pharmacokinetics, pharmacology, safety, industrial applications, and biosynthesis, with a particular focus on bridging basic science insights with translational and industrial perspectives.

A systematic search was conducted across three major electronic databases: PubMed, Web of Science and CNKI, covering publications from January 2005 to September 2025. The systematic search strategy was built around three core conceptual areas: (1) the plant and its key compounds, including terms such as “*Stevia rebaudiana* Bertoni,” “stevia,” “steviol glycosides,” “stevioside,” “rebaudioside A” (and its variants “Reb D,” “Reb M”), and “steviol”; (2) its biological and safety profile, using terms like “pharmacokinetics,” “metabolism,” “absorption,” “safety,” “toxicity,” “Acceptable Daily Intake (ADI),” “mutagenicity,” and “carcinogenicity”; and (3) its production and uses, with keywords such as “biosynthesis,” “glycosyltransferase,” “UGT76G1,” “sweetener,” “applications,” “functional food,” and “animal feed”. These terms were combined within and across categories using Boolean operators (AND/OR) to ensure a comprehensive retrieval of relevant literature. For example, to locate studies on the industrial use of stevia, search strings such as (“Stevia rebaudiana” OR “steviol glycosides”) AND (“applications” OR “functional food” OR “sweetener”) were used. The initial search across PubMed, Web of Science, and CNKI databases yielded a total of over 17,600 records before deduplication and screening. The detailed search strings and the corresponding hit counts for each key concept are provided in Supplementary Table S1. After removing duplicates and screening titles and abstracts for relevance, a pool of around 200 potentially eligible full-text articles was identified. To ensure comprehensiveness, additional relevant articles were identified through backward snowballing (scanning reference lists of key reviews and included studies) and forward citation tracking using Google Scholar. This supplementary search added roughly 50 key references. Given the narrative synthesis approach, article selection prioritized peer-reviewed research articles, authoritative reviews, and significant regulatory assessments (e.g., from EFSA, JECFA) that offered foundational insights, recent advances, or critical perspectives on the topics outlined in the review’s aims. Finally, more than 140 sources were selected for a rigorous evaluation and synthesis to construct this review. While this approach provides a broad and integrative overview, it is acknowledged that narrative syntheses may be influenced by the authors’ perspective in selecting and interpreting studies. Every effort was made to minimize this by focusing on high-impact and frequently cited evidence across the represented disciplines

## 3 Chemical components

The remarkable properties of stevia are rooted in its complex and unique chemical profile.

**FIGURE 1:**
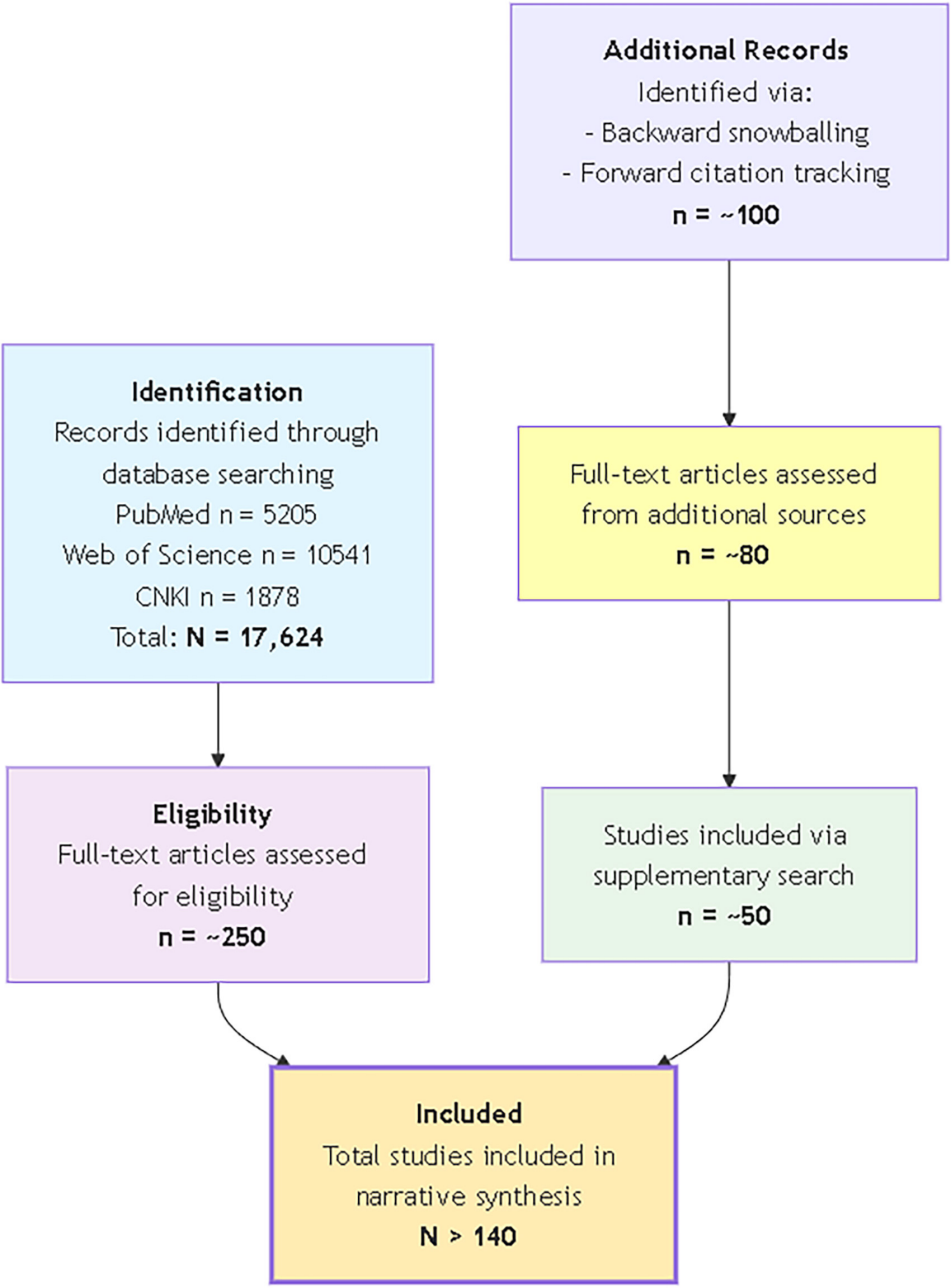
Flowchart of literature retrieval methods.

SGs are the most abundant and economically significant secondary metabolites in stevia leaves, accounting for approximately 4–20% of the dry leaf weight (28, 29). This wide variation is influenced by several factors, including the cultivar, cultivation conditions, fertilizer application, and harvest timing (29). These compounds are all derivatives of a single, common aglycone backbone (shown in Table 1 and Figure 1): steviol (a tetracyclic diterpenoid carboxylic acid) (**1**). The astounding diversity within SGs arises from the number, type, and linkage patterns of carbohydrate units attached to the two reactive sites of steviol: the C13-hydroxyl and the C19-carboxyl group (30).

**TABLE 1:** Chemical properties and sensory profiles of major SGs in *S. rebaudiana* leaves.

Compound (abbreviation)	Relative sweetness (vs. sucrose)	Sensory profile and aftertaste	Commercial and functional notes	References
STE	∼250–300 times	High sweetness, but accompanied by a noticeable bitter, licorice-like aftertaste.	Most abundant SG (22–62% of total SGs). The bitter aftertaste limits its standalone use in premium products.	(142)
Reb A	∼250–450 times	Cleaner sweetness than STE, with significantly reduced bitterness.	Widely used commercial standard due to its favorable taste profile. Content varies (5–65% of total SGs).	(26, 143, 144)
Reb C	∼20–30 times	No sweetness, moderate astringencye.	Reb C, which constitutes approximately 0.6% of wild stevia leaves and 1–2% of dry leaves. Its primary functions are to serve as an analytical standard in scientific research and to function as a flavor enhancer in blends with other SGs.	(144–147)
Reb D	∼200–220 times	Reduced bitterness and improved sweetness quality compared to Reb A, moving closer to a sucrose-like taste.	Characterized by its improved taste, Reb D constitutes 0.3–0.4% of stevia leaf dry matter and is a prioritized target for biosynthetic production.	(144, 148)
Reb M	∼200–350 times	Superior, clean, sugar-like sweetness. Lacks the characteristic bitter/licorice aftertaste of earlier-generation SGs.	Highly desired for its excellent taste but present in very low natural abundance (< 0.5%). Focus of modern biosynthetic production.	(26)
Dulcoside A	30 Times	Lower sweetness intensity and typically more pronounced bitterness.	A minor SG contributing to the overall bitter taste profile of crude extracts.	(149)
Steviol	Not sweet	Not sweet. The common metabolic product of all SGs formed by gut microbiota.	Not a sweetener. Its formation in the colon is key to the “zero-calorie” claim of SGs.	(42, 45)

The most prevalent SGs include STE (**2**), in which a glucose unit is esterified to the C-4 carboxyl group and a sophorose disaccharide (two glucose molecules linked via β(1–2) is attached at the C-13 position), along with various rebaudiosides (Reb A, B, C, D, E) (**3**–**7**) and dulcosides (**8**) (31). In native, non-selectively bred stevia cultivars, the SG composition varies considerably. Typically, STE constitutes 22–62% of the total SGs, while Reb A accounts for a smaller fraction, ranging from 5% to 22%. In contrast, improved cultivars developed through targeted breeding for high Reb A content can yield this glycoside at levels exceeding 25% of the total SG, with reported instances as high as 61.6%, surpassing the STE content in these improved varietie (32, 33). The number and type of glucose units, along with the nature of the connecting bonds, jointly determine the sensory properties of SGs, including sweetness, bitterness, and aftertaste. Generally, lengthening or modifying the sugar chain at the C-13 position, such as adding glucose units, enhances sweetness intensity. For instance, elongation of the ester bond at C-19—from Reb A to Reb D to Reb M—has been shown to significantly reduce the bitter, licorice-like aftertaste, yielding a purer sweet taste. Reb A, which has one additional glucose unit at C-13 compared to STE, serves as the basis for further derivatives, with Reb D and Reb M featuring additional glucose units at the C-19 position, resulting in superior sweet taste characteristics. Reb A is used commercially due to its clean sweet taste and minimal bitterness (34). Notably, Reb M (**9**) is considered the “new generation” of SG because it lacks the bitterness found in other SGs like Reb A and STE (35).

**FIGURE 2:**
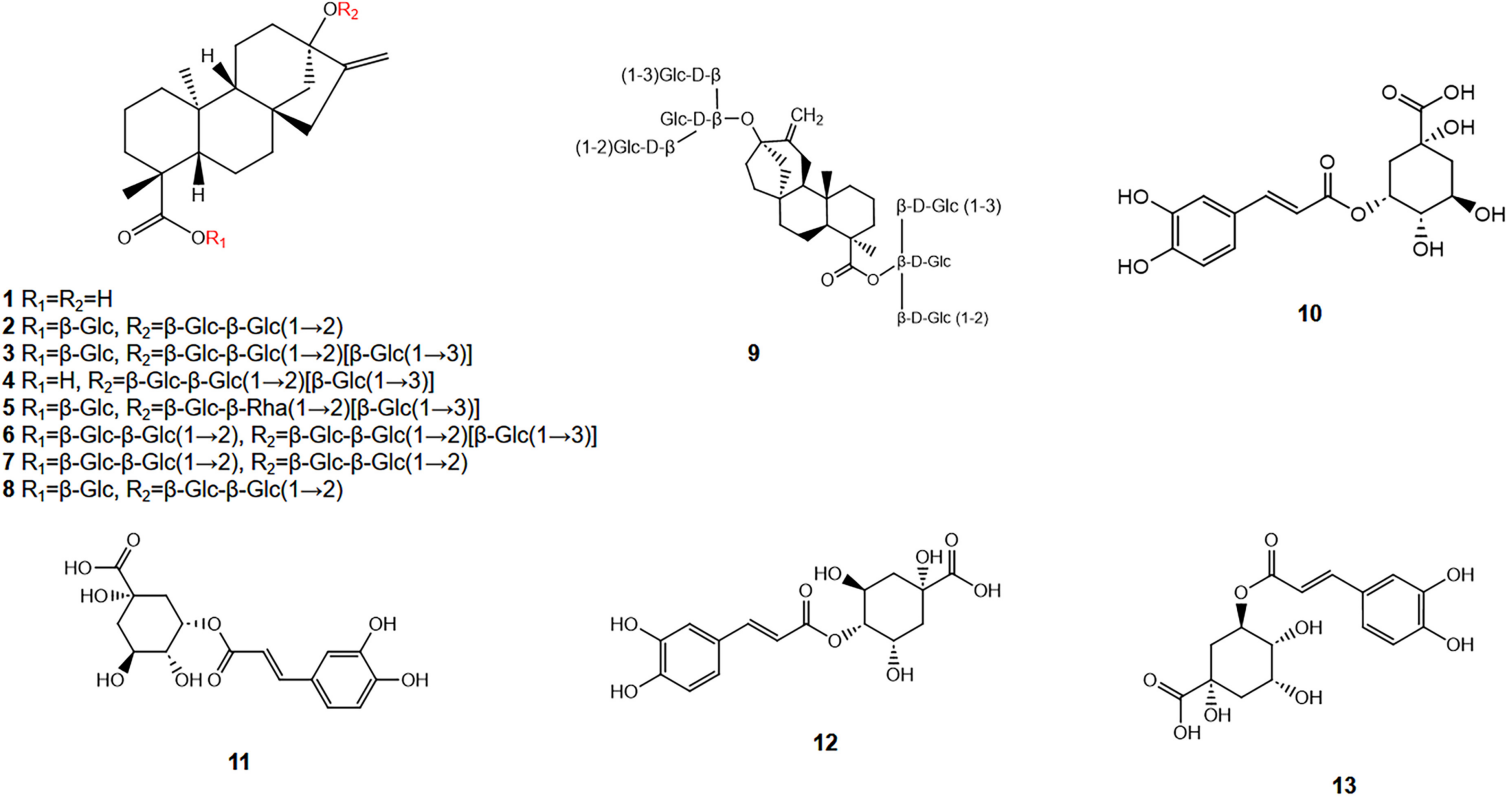
The chemical structure of the main glycosides in stevia leaves.

Stevia leaves contain a diverse array of bioactive compounds. Among these, phenolic compounds are significant secondary metabolites that exhibit elevated antioxidant activity, attributed to their multiple phenolic hydroxyl groups. The identified phenolic acids from the ethanol extract of stevia leaves include neochlorogenic acid (**10**), isochlorogenic acid (**11**), cryptochlorogenic acid (**12**), and chlorogenic acid (**13**) (shown in Figure 2). Among these four types of chlorogenic acids, isochlorogenic acid was the most abundant, constituting approximately 43.0–49.6 mg/g (4.30–4.96%) of the dry extract weight. The contents of the other isomers were as follows: neochlorogenic acid, 8.1–9.9 mg/g (0.81%–0.99%); cryptochlorogenic acid, 0.8–1.1 mg/g (0.08%–0.11%); and chlorogenic acid, 2.9–3.8 mg/g (0.29%–0.38%). Additionally, the phenolic acid profile encompassed caffeic acid and its derivatives, as well as quinic acid and its derivatives (36). Flavonoids constitute approximately 5 mg/g of the leaf dry weight, comprising rutin, quercetin, kaempferol, apigenin, and their respective derivatives (5, 6). Additionally, stevia leaves contain essential oils predominantly composed of sesquiterpenes, which, along with flavonoids and certain quinines, contribute to the characteristic bitter taste. Other components such as amino acids, alkaloids, vitamins, purines, and trace elements are also present (37). It is noteworthy that cultivation methods significantly influence the phytochemical profile of stevia: organically grown plants exhibit a markedly higher total phenolic content (0.948 ± 0.157 mg/g) than their conventionally cultivated counterparts (0.708 ± 0.089 mg/g), whereas the flavonoid levels are comparable between the two groups (0.165 ± 0.030 vs. 0.186 ± 0.088 mg/g) (38).

## 4 Pharmacokinetics and metabolic fate

### 4.1 A universal gateway: hydrolysis to steviol

Research on stevia metabolism has primarily focused on the native glycosides and key components such as STE and Reb A. However, studies have shown that both major and minor glycosides exhibit highly similar transformation patterns during microbial metabolism in the gut. Experiments have confirmed that a range of SGs, including STE, Reb A, Reb B, Reb C, Reb D, Reb E, Reb F, Reb M, dulcoside A, and STE dimer, are all completely hydrolyzed into the final product, steviol, by gut microbiota within 24–48 h. This metabolic process shows no significant differences based on gender or ethnicity (7, 8). These findings suggest that although different SGs vary in their glycosylation levels, their ultimate metabolic fate is highly consistent, with only slight variations in the hydrolysis rate (7, 8). This convergence explains why, from a toxicological and metabolic standpoint, the safety of all SGs can be evaluated based on the properties of their common metabolite, steviol.

Studies have demonstrated that SGs remain intact in the presence of digestive enzymes and gastric juices in both humans and rats, with minimal absorption occurring in the upper gastrointestinal tract (39, 40). However, in the lower gastrointestinal tract of rats, mice, pigs, and humans, SGs (including STE and Reb) can be metabolized by gut microbiota (particularly Bacteroides species) into steviol (8, 41).

Metabolic pathway analysis reveals that STE undergo initial hydrolysis to steviol by gut microbiota, followed by subsequent biotransformation in the hepatobiliary system and kidneys. Cytochrome P450 enzymes in the kidneys metabolize steviol into mono- and di-hydroxylated metabolites (42). The enterohepatic circulation facilitates the conjugation of steviol with glucuronic acid, forming steviol glucuronide, which is rapidly excreted in urine (43).

### 4.2 Subtle differences among human and rodents

**FIGURE 3:**
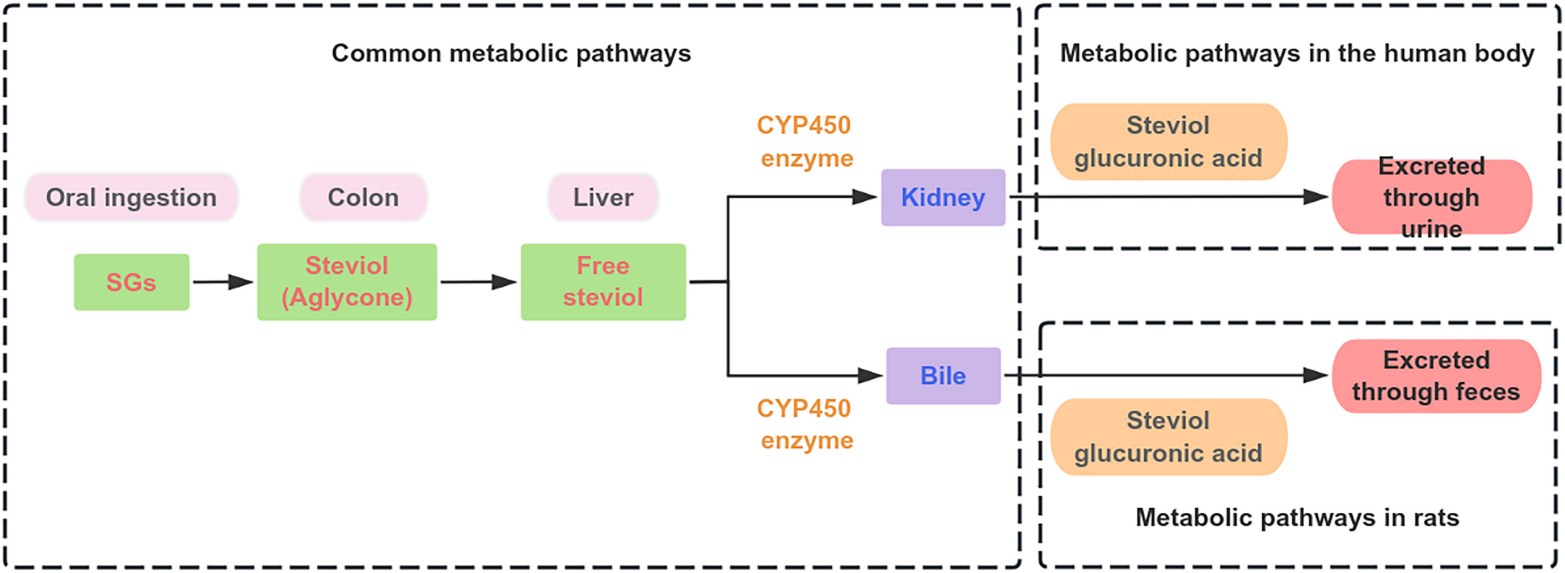
The metabolic pathways of SGs in rats and humans.

Although the ultimate metabolic fate is consistent, the kinetics and site of SGs uptake show weak differences (shown in Figure 3). In humans, STE was administered orally at high doses (750 mg⋅day^–1^), and neither the parent compound nor steviol was detected in the bloodstream. Steviol was exclusively identified in feces, indicating that absorption of the aglycone is minimal and that hydrolysis occurs primarily in the distal colon, with subsequent fecal excretion of unabsorbed steviol (43, 44). Rodents demonstrate a different pattern. While SGs are not absorbed, a portion of the liberated steviol is absorbed through the colonic epithelium into the portal vein. Subsequently, steviol undergoes extensive phase II metabolism (glucuronidation) in the liver to form steviol glucuronide, which is then excreted rapidly via bile back into the intestines, undergoing enterohepatic circulation, and is ultimately excreted predominantly in feces (42, 45).

### 4.3 The “zero-calorie” secret: microbes as the metabolic engineers

The claim of zero calories is directly attributable to this microbial-dependent metabolism. While the net metabolizable energy is not strictly “zero” from a thermodynamic standpoint, it is negligible under intended conditions of use. This aligns with regulatory definitions, such as China’s GB 28050-2011 standard, which permits a “zero-calorie” claim for solid/liquid foods containing ≤ 17 kJ per 100 g/mL (46).

The human body lacks the enzymes to break the glycosidic bonds in SGs, preventing their absorption in the small intestine where caloric uptake occurs. The sugar components released in the colon by bacterial hydrolysis are not absorbed by the host but are instead rapidly fermented by the gut microbiota as a preferential carbon source, producing short-chain fatty acids (acetate, propionate, and butyrate) (9). These short-chain fatty acids are then absorbed by the colonocytes, providing energy to the local gut tissue but contributing negligible net calories to the host systemic energy balance. This elegant mechanism is the cornerstone of SGs’ value as non-nutritive sweeteners. It is worth noting that “zero-calorie” applies only to highly purified SGs used as sweeteners, and does not necessarily apply to whole leaves or crude extracts that may contain other calorie-containing components.

### 4.4 Pathway of excretion

The final excretion pathway of metabolized SG derivatives highlights a key physiological distinction between humans and rodents. The primary metabolite, steviol glucuronide (molecular weight ∼512.9 Da), is water-soluble. In humans, compounds with a molecular weight below 600 Da are primarily excreted via the renal pathway, resulting in efficient elimination of steviol glucuronide in urine. In contrast, the biliary excretion threshold in rats is significantly lower (∼325 Da), leading to preferential excretion of steviol glucuronide via bile into the feces (43).

## 5 Pharmacological effects

**FIGURE 4:**
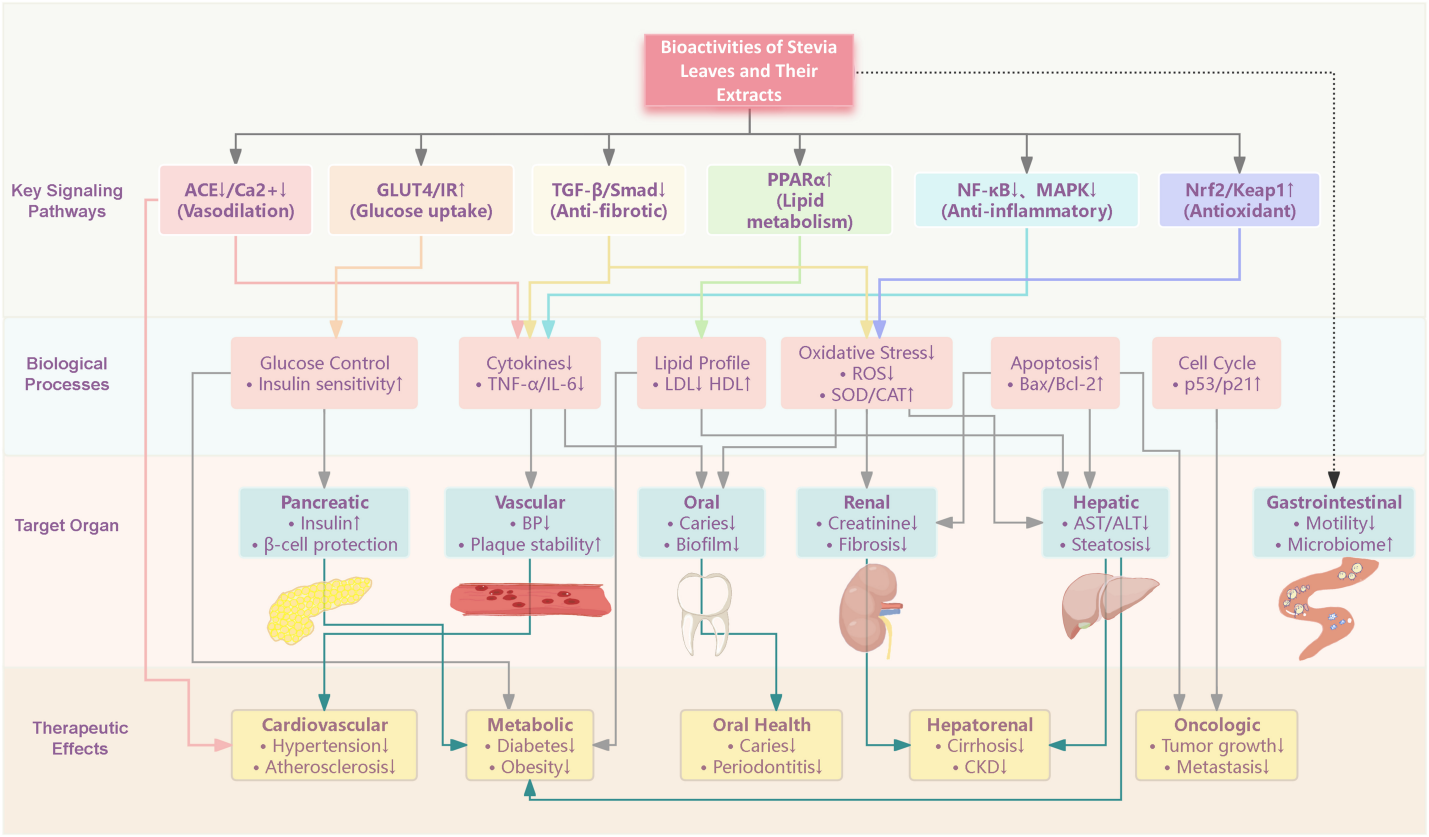
Pathological effects of the active components in stevia leaves and their extracts.

Various bioactive compounds in stevia have been experimentally shown to possess pharmacological effects, though most research has primarily focused on SGs like STE and Reb, which are key players in modulating critical pathways involved in oxidative stress, inflammation, metabolic dysregulation, and cell proliferation (shown in Figure 4 and Table 2). It is crucial to distinguish that the term “stevia” in research may refer to materials of varying composition and purity. Purified SGs (e.g., STE, Reb A) are defined single compounds. In contrast, extracts (e.g., alcoholic, aqueous, or residue extracts) are complex mixtures containing SGs along with other co-extracted compounds like flavonoids and phenolic acids (e.g., chlorogenic acid). The specific composition and observed bioactivity depend heavily on the extraction method and source material. For a detailed breakdown of these material categories, see Table 2 under “Material/Compound Type.”

**TABLE 2:** Pharmacological effects of *S. rebaudiana.*

Pharmacological effects	Types	Models	Number of samples	Material/ compound type	Doses of stevia	Effects/mechanism	References
Antioxidant	*In vitro*	SKVO3 cells	/	Stevia extract	Stevia hydromethanolic extract: 25, 50, and 100 μg/mL	Stevia hydromethanolic extract exerts potent antioxidant effects by significantly reducing intracellular ROS levels, thereby inhibiting lipid peroxidation, protecting erythrocytes from oxidative damage, and ultimately inducing apoptosis in ovarian cancer cells.	(47)
	*In vivo*	*Caenorhabditis elegans*	3	Pure SGs	Reb A: 1.67 mM	Reb A significantly attenuates aging in *Caenorhabditis elegans* by effectively reducing cellular ROS and inhibiting ectopic lipid accumulation, primarily via modulation of the TOR signaling pathway.	(48)
	*In vivo*	Weaned piglets	6	Stevia extract	Stevia residue extract: 100, 200, or 400 mg/kg/d for 42 days	Stevia residue extract (at 400 mg/kg) significantly enhanced the systemic and hepatic antioxidant capacity in weaned piglets, as evidenced by reduced oxidative stress marker (MDA) and elevated activity of key antioxidant enzymes (T-AOC, T-SOD, GSH-PX, CAT), while also modulating gut microbiota in a manner positively correlated with improved antioxidant status.	(49)
	*In vivo*	Layers	60	Chlorogenic acid	Stevia chlorogenic acid: 1, 2, and 4 g/L	Stevia-derived chlorogenic acid can enhance immune defense and alleviate oxidative stress-related inflammatory damage by regulating immune-related gene expression, improving intestinal barrier function, and modulating gut microbiota homeostasis in challenged organisms	(50)
	*In vivo*	High fat/low streptozocin-induced diabetic in rats	6	Pure SGs	STE: 12.5, 25, and 50 mg/kg/d for 21 days	STE alleviates oxidative DNA damage in the liver and kidneys of diabetic rats, primarily by reducing lipid peroxidation and nitric oxide levels, and its antioxidant mechanism is associated with the inhibition of beta-adrenergic receptor kinase and G-protein-coupled receptor kinase	(51)
	*In vivo*	D-galactose-induced aging in mice	5	Stevia extract	Stevia residue extract: 100, 200, and 500 mg/kg/d via gavage for 11 weeks	Stevia residue extract confers potent antioxidant protection by activating the Akt/Nrf2/HO-1 signaling pathway, thereby upregulating key endogenous antioxidant enzymes and reducing oxidative damage markers.	(52)
	*In vivo*	Cisplatin-induced kidney injury in mice	8	Stevia extract or pure SGs	Stevia ethanol extract or STE: 10, 20, and 50 mg/kg/d for 4 days	Stevia ethanol extract andSTE effectively attenuates cisplatin-induced nephrotoxicity by suppressing oxidative stress markers (4-hydroxynonenal, 3-nitrotyrosine, HO-1) and modulating downstream pro-apoptotic and inflammatory signaling pathways (ERK1/2, STAT3, NF-κB).	(53)
	*In vivo*	/	/	Stevia leaves extract	0.5 g powdered dry leaves or 1 g fresh leaves mixed in a 1:10 ratio with ultra-pure water	The drying method critically modulates the functional profile of stevia leaves, with freeze- and shade-drying optimally preserving antioxidant polyphenols, while microwave-drying yields extracts with the strongest anti-inflammatory activity.	(54)
Antiinflammation	*In vivo*	Obesity and insulin resistance mice	8	Pure SGs	STE: 10 mg/kg/d for 12 weeks; Reb A: 12 mg/kg/d for 12 weeks; steviol: 5 mg/kg/d for 12 weeks	STE, Reb A, and steviol exert significant hepatoprotective effects by attenuating hepatic steatosis through multifaceted mechanisms, including the modulation of PPAR-regulated pathways, which collectively improve glucose and lipid metabolism, reduce inflammation, and mitigate oxidative stress.	(55)
	*In vivo*	Obesity and insulin resistance mice model	14	Pure SGs	STE: 10 mg/kg/d for 12 weeks	STE exerts its anti-inflammatory and anti-atherosclerotic effects primarily by enhancing insulin signaling and systemic antioxidant defense, which collectively increase adiponectin levels and promote the stabilization of vascular plaques.	(56)
	*In vivo*	LPS-stimulated colonic epithelial cells	/	Pure SGs	STE: 0.001–1 mmol L^–1^; steviol: 0.1–100 μmol L^–1^	STE and steviol attenuate LPS-induced pro-inflammatory cytokine productions by affecting cytokine gene expression via IκBα/NF-κB signaling pathway	(57)
	*In vivo*	*Staphylococcus aureus*-infected mouse mammary epithelial cells	/	Pure SGs	STE: 33, 100, and 300 μg/mL for 1 h	STE exerts its anti-inflammatory effects by dose-dependently inhibiting the release of pro-inflammatory cytokines (TNF-α, IL-6, IL-1β) and suppressing the activation of key signaling proteins in the TLR2/NF-κB/MAPK pathways	(58)
	*In vivo*	TAA-induced cirrhosis in rats	8	Stevia extract	Stevia aqueous extract: 100 mg/kg/d over 10 weeks	Stevia demonstrated potent anti-inflammatory, antioxidant, and antifibrotic effects in a rat model of cirrhosis by upregulating the protective Nrf2 pathway, downregulating the pro-inflammatory NF-κB pathway, and subsequently inhibiting hepatic stellate cell activation.	(60)
	*In vivo*	STZ and nicotinamide-induced diabetic in rats	8	Pure SGs	Stevia: 400 mg/kg for 3 weeks	Stevia not only exerts an antihyperglycemic effect by lowering blood glucose, improving lipid profiles, and reducing oxidative stress, but also synergistically enhances the efficacy of the antidiabetic drug saxagliptin, likely through mechanisms involving DPP-4 inhibition and improved insulin sensitivity.	(61)
	Human	T2DM patients	15	Stevia extract	Received 1 cup of 2% stevia extract sweet tea in three meals for 8 weeks	Consumption of stevia aquatic extracStevia alleviates hyperglycemia and associated kidney injury in diabetic rats by upregulating key glucose transporters (GLUT-4, SNAP23, Stx4) in skeletal muscle and enhancing the renal antioxidant defense pathway (Nrf2/Keap1), showing superior efficacy over metformin in increasing GLUT4 and Nrf2 mRNA expression. t is associated with a significant reduction in blood glucose levels, particularly at higher dosages and within a short to medium term, though it does not significantly impact insulin concentration or HbA1c levels.	(62)
Anti- hyperglycem Ia	*In vivo*	STZ and nicotinamide-induced diabetic in rats	10	Stevia extract	Stevia aqueous extract: 400 mg/kg/d over 30 days	Stevia alleviates hyperglycemia and associated kidney injury in diabetic rats by upregulating key glucose transporters (GLUT-4, SNAP23, Stx4) in skeletal muscle and enhancing the renal antioxidant defense pathway (Nrf2/Keap1), showing superior efficacy over metformin in increasing GLUT4 and Nrf2 mRNA expression	(11)
Anti-hyperglycemia	*In vivo*	STZ -induced diabetic in rats	8	Stevia extract	Stevia aqueous extract: 400 mg/kg/d for 28 days	Stevia aqueous extract exerts anti-hyperglycemic effects by elevating pancreatic insulin levels via a PPARγ-dependent mechanism, while its antioxidant properties contribute to mitigating diabetes-associated metabolic and tissue damage.	(64)
	*In vivo*	Insulinoma MIN6 cells	4/6/12	Pure SGs	STE and Reb A: 10–6 M	The anti-hyperglycemic action of Reb A is achieved by a glucose-dependent inhibition of ATP-sensitive K^+^ channels, likely mediated through increasing the ATP/ADP ratio, which promotes insulin secretion while potentially posing a lower risk of hypoglycemia compared to sulfonylureas.	(65)
	*In vivo and in vitro*	*In vivo:* Trpm5^–/–^ mice *in vitro:* HEK293T and HEK cells	mice: *n* = 6; cells: *n* = 4	Pure SGs	*In vivo:* STE, Reb A, and steviol 100 mg/L/d for 10 or 15 weeks; *in vitro:* STE, Reb A, and steviol (10 μM)	STE, Reb A, and steviol by potentiating the TRPM5 channel, which enhances sweet taste perception and, more importantly, glucose-stimulated insulin secretion, thereby effectively preventing high-fat-diet-induced hyperglycemia.	(10)
Anti-hyperlipidemic	*/*	Protein active site evaluation	/	Pure SGs	STE	STE exhibits potent anti-hyperglycemic effects by directly and stably binding to key targets in the insulin signaling pathway (GLUT4, Akt, IR, IRS-1) to promote glucose uptake in muscles, alongside its established benefits of lowering blood sugar and pressure, thereby presenting a safe and promising dietary adjunct for diabetic management.	(39)
	*In vivo and in vitro*	*In vivo:* db/db mice *in vitro:* HepG2 cells	6	Stevia leaves extract	*In vivo:* stevia leaves extract: 200 and 500 mg/kg/d for 3 weeks; STE: 40 mg/kg/d for 3 weeks; *in vitro:* STE: 2.5, 25, 50, and 100 μM	Stevia and its componentSTE alleviate hepatic steatosis and hyperlipidemia in db/db mice by activating PPARα-dependent lipophagy, thereby reducing body/liver weight and serum lipid levels.	(12)
Anti- hyperlipide mic	*In vivo*	Mature spontaneously hypertensive rats	10	Stevia leaves extract	Stevia leaf ethanol extract: mixed with 1.0 g stevia leaf powder in 10 mL aqueous ethanol solution	Stevia leaf ethanol extract exhibit potent, dose-dependent ACE inhibitory activity.	(14)
	*In vivo*	STZ -induced diabetic in rats	8/9/10	Pure SGs	STE or Reb A: 500 or 2,500 mg/kg/d for 5 weeks	STE or Reb A normalizes hyperlipidemia and alleviates tissue damage in diabetic rats, without significantly affecting blood glucose or insulin resistance	(67)
	*In vivo*	Albino rats	10	Stevia extract	Stevia aqueous extract 200, 300, 400, and 500 ppm/kg/d for 8 weeks	Stevia aqueous extract exerts a significant anti-hyperglycemic effect by improving lipid profiles, notably by reducing LDL-cholesterol and increasing HDL-cholesterol in hyperlipidemic rats.	(68)
	Human	Chinese hypertensive subjects	106	Pure SGs	Capsules containing STE (250 mg) thrice daily for 1 years	STE (750 mg/day) effectively and safely lowers both systolic and diastolic blood pressure in hypertensive patients over a 1-year period without adversely affecting blood glucose or lipid profiles.	(69)
Anti-hypertension	Human	Chinese men and women between the ages of 20 and 75 years with newly diagnosed mild (stage 1) essential hypertension	174 (87 Men, 87 women)	Pure SGs	STE capsules 500 mg/d for 2 years	STE significantly lowers both systolic and diastolic blood pressure while improving quality of life, without inducing significant adverse effects, in patients with mild hypertension	(70)
	*In vivo*	Male wistar rats	10	Stevia extract	Stevia extract: 0.05 mg/min/100 g	Stevia extract exerts its antihypertensive effect not by directly lowering systemic arterial pressure, but by promoting renal water and sodium excretion through preferential action on the salt transport mechanisms in proximal tubular cells.	(13)
Anti-caries and antimicrobial	*In vivo*	Albino sprague-dawley rats	8	Pure SGs	STE and Reb A: 0.5% w/w	The STE and Reb A do not promote dental caries or increase cariogenic *Streptococcus sobrinus* colonization in a rat model, demonstrating their non-cariogenic potential.	(74)
	*In vivo*	*Porphyromonas gingivalis* infected periodontitis mice models	6	Pure SGs	0.1% w/w STE	STE demonstrates significant anti-caries and antimicrobial potential by suppressing key periodontal pathogens (e.g., *Porphyromonas gingivalis*), reducing inflammatory cytokine production, inhibiting alveolar bone resorption, and promoting a healthier oral bacterial profile.	(19)
	*In vivo*	Dental caries of premolar enamel	9	Stevia extract	20 Percent solution of water/methanol extract of stevia	Stevia possess significant anti-caries potential by effectively reducing enamel demineralization depth compared to conventional sugars like glucose and fructose.	(75)
	*In vivo*	Dental caries	*Streptococcus* (*n* = 12); *Lactobacillus* (*n* = 4)	Stevia leaves extract	Stevia leaves extracts: Hexane 30 mg/mL; Methanol 120 mg/mL; Ethanol 120 mg/mL; Ethyl acetate 60 mg/mL; Chloroform 60 mg/mL	This study reveals that various leaf extracts of stevia, particularly the hexane extract with a low minimal inhibitory concentration (MIC of 30 mg/mL), exhibit direct antibacterial activity against key cariogenic bacteria (*Streptococcus* and *Lactobacillus* spp.).	(76)
	*In vitro*	Two-species biofilm model of *Streptococcus mutans* and *Candida albicans*	/	Pure SGs	STE: 1 g/100 mL	STE demonstrates significant anti-caries and antimicrobial efficacy by inhibiting the growth, acid production, and virulence of cariogenic pathogens (*Streptococcus mutans* and *Candida albicanss*) within dual-species biofilms, while also suppressing biofilm formation and reducing fungal pathogenicity.	(18)
	*In vitro*	TPA-induced two-stage carcinogenesis in mice skin	15	SGs mixture	SGs mixture (STE, Reb A, Reb C, and dulcoside A): 1.0 and 0.1 mg/mouse	SG smixturedemonstrated significant chemopreventive potential by suppressing both TPA-induced inflammation and tumor promotion.	(77)
	*In vitro*	PA induced EBV-EA activation in Raji cells	/	Pure SGs	STE, isosteviol, and its 5 metabolites (T-hydroxyisosteviol, 7-oxoisosteviol, 11β-hydroxyisosteviol, 12β-hydroxyisosteviol, 17-hydroxyisosteviol)	STE, isosteviol, and its 5 metabolites (T-hydroxyisosteviol, 7-oxoisosteviol, 11β-hydroxyisosteviol, 12β-hydroxyisosteviol, 17-hydroxyisosteviol) can inhibit swelling tumor promoter TPA induced EBV-EA activation in Raji cells was inhibited, and five metabolites showed stronger inhibitory effects.	(78)
Anti-tumor	*In vitro*	Bladder cancer cell lines (T24, 5,637) and normal urothelial cell line (SV-HUC-1)	/	Pure SGs	STE: 10, 20, and 40 μM	STE exerts anti-tumor effects by selectively inducing bladder cancer cell apoptosis through a ROS-driven, GSK-3β-mediated mechanism that converges on mitochondrial stress and ER stress, ultimately downregulating Mcl-1 and upregulating Noxa to activate Bax.	(20)
	*In vitro*	Human gastrointestinal cancer cell lines (Caco-2, HCT-8, HCT-116, MKN-45, MGC-803, and HGC-27)	/	Pure SGs	Steviol: 50, 100, and 200 μg/mL	Steviol exerts potent anti-tumor effects on gastrointestinal cancer cells by inducing mitochondrial apoptosis and modulating specific microRNA expression.	(79)
	*In vitro*	Human breast cancer cell line (MCF-7)	/	Pure SGs	Steviol: 185 μg/mL	Steviol exerts anti-tumor activity against MCF-7 breast cancer cells by inducing dose-dependent apoptosis and causing cell cycle arrest at the G2/M phase	(80)
	*In vitro*	Breast cancer cell line (MCF-7 and MDA-MB-231)	/	Pure SGs	SGs: MCF-7 (10, 25, and 40 μM); MDA-MB-231 (100, 250, and 400 μM)	SGs exhibit a context-dependent dual role in anti-tumor activity, consistently inducing apoptosis across breast cancer cell lines while differentially modulating metastatic behaviors—potentially promoting migration in less aggressive subtypes but significantly inhibiting it in highly metastatic cells.	(81)
	*In vitro*	Human cancer cells	/	Pure SGs	Isosteviol: 84–167 μM	Steviol exerts its anti-tumor potential by selectively inhibiting key human DNA replication and repair enzymes (DNA polymerases and topoisomerase II), thereby suppressing the proliferation of cancer cells.	(82)
	*In vitro*	Enterohemorrhagic *Escherichia coli* O157	/	Stevia extract	Stevia hot-water extract:10, 20, and 30%	Stevia extract exerts anti-diarrheal potential by demonstrating strong, pH-dependent bactericidal activity against key enteric pathogens like *Escherichia coli* O157:H7 while sparing beneficial gut microbiota such as *Bifidobacteria* and *Lactobacilli*.	(83)
	*In vitro*	HRV-infected MA104 cells	/	Pure SGs	STE or stevia: 500 mg/mL	The hot water extract of stevia demonstrates potent anti-human rotavirus (HRV) activity by specifically binding to the viral VP7 protein, thereby blocking viral attachment to host cells and inhibiting replication across all four HRV serotypes.	(84)
Anti- diarrhea	*In vitro*	The smooth muscle of isolated guinea pig ileum	/	Stevia extract	Stevia hot-water extract: 0.1, 0.5, and 1.0 mg/mL	A hot water extract of stevia exhibits anti-diarrheal potential by non-specifically antagonizing smooth muscle contractions in the isolated guinea pig ileum, an effect attributed toSTE and associated with the inhibition of extracellular Ca^2+^ influx.	(85)
	*In vitro*	Laying hens	8	Stevia extract	Stevia extract: 50, 100, 200, and 400 mg/kg	Stevia extract (at an effective level of 200 mg/kg) can confer anti-diarrheal benefits in laying hens by improving gut health, as evidenced by the enhancement of intestinal structure (increased villus height/crypt depth ratio), modulation of the cecal microbiota (increased alpha diversity and altered Bacteroidetes/Firmicutes abundance), and boosted systemic immunity.	(87)
	*In vivo*	Weaned piglets	6	Pure SGs	STE and Reb A: 1,100, 150, 200, 250, and 300 mg/kg	Dietary supplementation with STE or Reb A significantly reduces diarrhea incidence in weaned piglets, with optimal anti-diarrheal effects observed at doses of approximately 200–250 mg/kg for STE and 191–213 mg/kg for Reb A	(88)
	*In vivo*	LPS-induced acute liver injury rats model	8	Stevia leaves extract	Stevia leaves hydroalcoholic extract: 500 mg/kg p.o; STE: 250 mg/kg p.o	Stevia extract and STE provide significant hepatoprotection against acute liver injury by effectively reducing oxidative stress and suppressing the hepatic inflammatory response	(89)
Protect the liver and kidneys	*In vivo*	STZ-induced diabetic model in male Wistar rats	8	Pure SGs	Stevia and nano-stevia: 20 mg/dl for 30 days daily	Both stevia and its nano-niosome formulation exert significant hepatoprotective effects in diabetic rats by concurrently reducing hepatic inflammation, oxidative stress, fibrosis, and enhancing glucose transporter (GLUT-2/9) expression.	(90)
	*In vivo*	CCl_4_-induced cirrhosis in rats	8	Stevia extract	Stevia aqueous extract: 100 mg/kg by gavage daily for 12 weeks	Stevia demonstrates hepatoprotective effects against CCl_4_-induced liver injury by counteracting oxidative stress, suppressing inflammation, and inhibiting hepatic stellate cell activation and fibrogenesis, primarily through the upregulation of the Nrf2 pathway and downregulation of NF-κB and profibrogenic signaling.	(91)
	*In vivo and in vitro*	*In vivo:* db/db mice *in vitro:* HepG2 cells	6	Stevia leaves extract and pure SGs	*In vivo:* stevia leaf extract: 200 and 500 mg/kg for 3 weeks; STE: 40 mg/kg for 3 weeks; *in vitro:* STE: 2.5, 25, 50, and 100 μM	Stevia and its component STE alleviates hepatic steatosis by activating PPARα-dependent lipophagy, thereby demonstrating hepatoprotective potential through enhanced lipid breakdown and clearance.	(12)
	Human	Stage II CKD patients	9	Pure SGs	Stevia capsule: 250 mg twice daily	Stevia supplementation significantly improved key renal and metabolic parameters (serum creatinine, uric acid, blood glucose, and microalbuminuria) in patients with CKD, suggesting its hepatorenal protective potential.	(92)
	*In vivo*	Gentamycin-induced nephrotoxicity in rat	6	Pure SGs	Stevia: 200 mg/kg/day; p.o. for 30 days	Stevia exhibits significant renoprotective effects in a rat model of drug-induced kidney injury, comparably attenuating serum creatinine elevation and ameliorating renal tissue damage, with its efficacy positioned between the superior valsartan and the potentially detrimental losartan	(93)
	*In vivo*	STZ -induced diabetic in rats	10	Stevia leaves extract	4.0% Stevia leaves powder incorporated diet (4.0 g leaf powder in 96 g dry diet) for 30 days	Stevia leaves and their polyphenols can concurrently protect against liver and kidney damage in diabetic rats, not only by improving glycemic control but also by enhancing antioxidant defenses, reducing hepatic oxidative stress markers, and ameliorating the decline in renal glomerular filtration rate.	(94)
	*In vivo*	Adenine-induced chronic kidney disease in mice	10	Stevia extract	Stevia residue extract: 200 and 400 mg/kg for 3 weeks	Stevia residue extract exerts a renoprotective effect in a mouse model of chronic kidney disease by ameliorating renal functional and structural damage, mitigating fibrosis through inhibition of the TGF-β1/Smad and Wnt/β-catenin pathways, and positively modulating the gut microbiota composition	(95)
	*In vivo*	STZ and nicotinamide-induced diabetic rats	10	Stevia extract	Stevia aqueous extract: 400 mg/kg over 30 days	Stevia may protect the kidneys in diabetic conditions by upregulating antioxidant (Nrf2/Keap1) and water transport (AQP2) pathways, and by improving skeletal muscle glucose uptake (GLUT-4, SNAP23, Stx4), thereby alleviating diabetic nephropathy.	(11)

### 5.1 Antioxidant

Oxidative stress, characterized by an imbalance between reactive oxygen species (ROS) and antioxidant defenses, is a fundamental pathological state underlying numerous chronic diseases.

Extensive research has demonstrated the potent antioxidant activity of stevia. A meta-analysis incorporating 184 studies and 104 datasets from healthy animals, diseased rats, and STE-treated rats revealed that stevia leaf extracts significantly outperformed pure SGs in restoring oxidative stress markers—such as superoxide dismutase (SOD), catalase (CAT), glutathione peroxidase (GPX), malondialdehyde (MDA)—by 65–85%, particularly in diabetic rat models (17). *In vitro* studies corroborated these findings, showing that stevia hydromethanolic extracts (12.5–400 μg/mL) protect erythrocytes from oxidative damage and induce apoptosis in SKOV3 ovarian cancer cells by reducing ROS levels and inhibiting cell migration and invasion (47). Additionally, Reb A (0.0083, 0.017, 0.033, and 0.05 mM) has been shown to enhance oxidative stress resistance in *Caenorhabditis elegans*, extending lifespan through ROS reduction (48).

Stevia’s chlorogenic acid (isolated from stevia leaves) also exhibits notable antioxidant properties. The addition of stevia residue extract, containing over 400 mg/kg chlorogenic acid, to weaned piglet feed not only reduces diarrhea rates but also improves antioxidant capacity, promoting piglet health (49). Furthermore, adding 2 g/L of stevia chlorogenic acid to the drinking water of egg chicks can alleviate oxidative stress induced by *Escherichia coli* O78, enhance intestinal barrier function, boost immunity, and reduce mortality in the chicks (50).

Mechanistic studies suggest that the antioxidant effects of stevia’s active components, such as STE (12.5, 25, and 50 mg⋅kg^–1^⋅day^–1^), may involve inhibition of β-adrenergic receptor kinase and G protein-coupled receptor kinase (51). Stevia residue extracts (200 mg⋅kg^–1^) protect against D-galactose-induced oxidative stress in aging mice through protein kinase B (Akt)/nuclear factor erythroid 2-related factor 2 (Nrf2)/enzyme heme oxygenase-1 (HO-1) pathway activation (52). Both ethanolic extracts (10, 20, and 50 mg⋅kg^–1^) and STEs (50 mg⋅kg^–1^) mitigate renal oxidative stress in mice by suppressing extracellular regulated extracellular signal-regulated kinase (ERK)1/2, signal transducer and activator of transcription 3 (STAT3), and nuclear factor kappa B (NF-κB) pathways, reducing free radical toxicity (53). Additionally, Reb A (0.0083, 0.017, 0.033, and 0.05 mM) may exert cellular antioxidant effects and reduce ectopic lipid accumulation through autophagy activation via target of rapamycin (TOR) and phosphatidylin-ositol-3-kinase (PI3K)/Akt signaling pathway inhibition (48).

Despite these promising findings, current research primarily focuses on the antioxidant mechanisms of stevia’s main active components, such as STE and Reb A. The mechanism of other compounds, like chlorogenic acid and isochlorogenic acid, remain underexplored. Future research should aim to investigate these lesser-studied components in more detail, offering a fuller understanding of stevia’s antioxidant potential. Additionally, studies on the synergistic effects of different stevia compounds could provide valuable insights into their combined therapeutic applications.

### 5.2 Anti-inflammation

Inflammation is a natural protective response that helps the body defend against external damage and pathogens. The activation of the innate immune system triggers the release of pro-inflammatory mediators, which recruit immune cells to the site of injury or infection. For example, interleukins (IL), a type of pro-inflammatory mediator, mobilize immune cells to target pathogens, foreign substances, and even cancer cells. However, when inflammation becomes uncontrolled and excessive pro-inflammatory mediators are produced, it can lead to a range of acute and chronic diseases. Chronic inflammation is a key driver of various conditions, including autoimmune disorders and metabolic syndrome-related diseases, such as atherosclerosis, obesity, fibrosis, and cancer.

Stevia has demonstrated anti-inflammatory properties, though its effectiveness can vary depending on the drying method used. stevia processed through freezing, convective drying, shade drying, vacuum drying, microwave drying, infrared drying, and sun drying can all mitigate the inflammatory response in mice induced by arachidonic acid to varying degrees. Among these, vacuum drying and microwave drying yield the most significant effects. Regarding the reduction of inflammation induced by phorbol 12-O-tetradecanoylphorbol-13-acetate (TPA), stevia leaf extracts (3 mg⋅ear^–1^) from microwave-treated, sun-dried, and shade-dried stevia demonstrated the best outcomes when exposed to TPA (5 μg in 20 μL acetone) or arachidonic acid (AA) (2 mg in 20 μL acetone) (54). Although STE (10 mg⋅kg^–1^⋅day^–1^), Reb A (12 mg⋅kg^–1^⋅day^–1^), and steviol (5 mg⋅kg^–1^⋅day^–1^) vary in their degrees of improvement in glycolipid metabolism, inflammation, and oxidative stress, they exhibit similar efficacy in alleviating hepatic steatosis in ob/ob (genetically modified mice with a leptin gene mutation leading to obesity) and low density lipoprotein (LDL) receptor-deficient mice (55). Furthermore, from a clinical perspective, the anti-inflammatory benefits of stevia may translate into a reduced risk of cardiovascular diseases. Research indicates that mice receiving STE (10 mg⋅kg^–1^) exhibit improved atherosclerosis and more stable plaques, likely due to increased circulating adiponectin levels (56).

From a mechanistic standpoint, both *in vitro* (with STE concentrations ranging from 0.001 to 1 mmoL/L and steviol from 0.1 to 100 μmoL/L) and *in vivo* (using STE at concentrations of 30, 100, or 300 μg/mL) experiments have revealed that the active components of stevia may modulate cytokine gene expression via the mitogen-activated protein kinase (MAPK) and inhibitor kappa B alpha (IκBα)/NF-κB signaling pathways, thereby reducing pro-inflammatory cytokine production (57, 58). On the other hand, Reb A (20 mg⋅kg^–1^, administered intraperitoneally twice daily) and the aqueous extract of stevia (100 mg⋅kg^–1^, administered daily by gavage) may enhance Nrf2-mediated antioxidant defenses. Subsequently, they may also inhibit the dual regulation of NF-κB and transforming growth factor β1 (TGF-β1)/SMAD family member (Smad) 7/Smatrix metalloproteinase (MMP)-13, working synergistically to alleviate oxidative stress and exert anti-inflammatory effects (59, 60).

### 5.3 Anti-hyperglycemia

Diabetes is a prevalent chronic metabolic dysbiosis. Type 1 diabetes mellitus primarily results from the destruction of pancreatic beta cells, whereas T2DM arises from the diminished responsiveness of body cells to insulin, leading to ineffective insulin utilization. A substantial body of research indicates that stevia and its extracts hold significant value in the management of T2DM. Various forms of stevia extract—including an aqueous extract (400 mg⋅kg^–1^⋅day^–1^), a standardized extract (2% w/w), and the SGs (≥ 3,342 mg⋅day^–1^)—have shown promising effects in regulating blood glucose, improving insulin sensitivity, and protecting β cells, without significantly altering HbA1C levels (61–63).

The hypoglycemic mechanisms of stevia involve several pathways: Stevia aqueous extract (400 mg⋅kg^–1^) regulates glucose transporter 4 (GLUT4), synaptosomal-associated protein 23 (SNAP23), and synaptosomal protein 4 (STX4) in skeletal muscle to lower blood glucose (11). Stevia aqueous extract (400 mg⋅kg^–1^) also acts through a peroxisome proliferator-activated receptor (PPAR) γ-dependent pathway in the pancreas to exert its anti-diabetic effects (64). In hyperglycemic conditions, insulin secretion shows a dose-response relationship with Reb A and SGs. Reb A (10^–9^ M) and STE (10^–6^ M) reduce the conductance of adenosine triphosphate (ATP)-sensitive potassium channels in a glucose-dependent manner (65). Additionally, STE, Reb A, and steviol (25 mg⋅kg^–1^ in a 0.1% solution in water) activate calcium ion (Ca^2+^), which in turn activate taste receptors and the transient receptor potential cation channel subfamily M member 5 (TRPM5) on pancreatic β-cells, thereby enhancing glucose-induced insulin secretion and improving the body’s blood glucose status (10). Some studies have also discovered that steviol (1 mM) can inhibit intestinal glucose absorption by reducing ATP content in the intestinal mucosa and altering the morphological structure of the intestine *in vitro* (66). Recent molecular docking studies suggest that STE may further improve glycemic control and reduce blood pressure by interacting with GLUT4, Akt, the insulin receptor (IR), and insulin receptor substrate-1 (IRS-1), promoting muscle glucose uptake (39).

### 5.4 Anti-hyperlipidemic

Emerging evidence highlights the potential of stevia as natural anti-obesity and lipid-regulating agents. In animal models, after db/db (leptin receptor point mutations lead to leptin signaling pathway dysfunction) mice were administered stevia leaf extracts (200 and 500 mg⋅kg^–1^⋅day^–1^) and STE (40 mg⋅kg^–1^⋅day^–1^) for 3 weeks, their body weight, liver weight, and serum lipid profiles, including triglycerides (TG), total cholesterol (TC), low-density lipoprotein (LDL) significantly decreased (12). A study explored the anti-hyperlipidemic effects of aqueous stevia extract (200, 300, 400, and 500 ppm kg^–1^ body weight) in albino rats with induced hyperlipidemia (400 mg⋅kg^–1^ body weight of cholesterol). The results demonstrated significant reductions in cholesterol, triglycerides, LDL, and VLDL, as well as an increase in HDL, highlighting stevia’s potential as a nutraceutical for managing hyperlipidemia and its complications (14). Similarly, Wistar rats fed high-fat diets supplemented with pure STE or Reb A at 500 or 2,500 mg⋅kg^–1^ body weight for 5 weeks normalized hyperlipidemia, improved appetite regulation, and alleviated tissue damage (67). In diet-induced hyperlipidemic albino rats, aqueous extract of stevia (200–500 ppm/kg b.w.) for 8 weeks significantly reduced body weight gain, TC, TG, LDL, very-low-density lipoprotein (VLDL), and LDL/high-density lipoprotein (HDL) ratios while increasing HDL levels (68). Mechanistically, the LDL-lowering effect of SGs is attributed to their ability to upregulate LDL receptors, enhancing cholesterol clearance from the blood (68).

### 5.5 Anti-hypertension

Numerous scientific studies have extensively explored the potential relationship between stevia and reduced blood pressure levels in both animal models and human populations. STE (250 mg three times daily for 1 year; 500 mg three times daily for 2 years) has demonstrated effective antihypertensive effects in rats, dogs, and humans (69–71). Two double-blind clinical trials involving Chinese patients with mild hypertension, lasting 1 and 2 years, respectively, found that oral administration of STE (250 mg three times daily for 1 year; 500 mg three times daily for 2 years) significantly reduced systolic and diastolic blood pressure compared to a placebo, with no significant adverse effects reported (69, 70). However, Reb A (500, 700, 1,000 mg⋅d^–1^) showed no significant effects on blood pressure or cardiovascular risk factors (72).

Mechanistically, intravenous infusion of stevia extract (0.05 mg/min/100 g) can effectively lower blood pressure in hypertensive rats by inducing systemic vasodilation and promoting natriuresis (13). The antihypertensive effect of STE may be linked to its significant an ACE inhibitory activity. Additionally, the vasodilatory effect of STE is at least partially attributed to its ability to inhibit Ca^2+^ influx into arterial vascular smooth muscle cells (73).

To unlock stevia’s full therapeutic potential as a natural antihypertensive agent, future research should focus on several key areas. First, molecular studies should clarify stevia’s ACE inhibitory activity and vasodilation mechanisms, including its interactions with cardiovascular pathways, to better understand its effects on hypertensive individuals. Second, long-term safety evaluations in populations with comorbidities (e.g., diabetes, kidney issues) are essential to ensure broader applicability and minimize risks. Third, large-scale clinical trials across diverse populations are needed to validate stevia’s antihypertensive effects and ensure its universal efficacy and safety. Fourth, research should explore stevia’s synergy with existing antihypertensive medications to improve blood pressure management. Finally, targeted studies should assess stevia’s efficacy in specific populations, such as those with refractory hypertension or high cardiovascular risk, to maximize clinical impact.

### 5.6 Anti-caries and antimicrobial

The formation of dental caries results from the complex interactions among acid-producing bacteria, fermentable carbohydrates, and many host factors (such as teeth and saliva). Das et al. tested the cariogenicity of STE and Reb A in *Streptococcus sobrinus-*infected Sprague-Dawley rat pups divided into four diet groups: 30% sucrose, 0.25% STE, 0.5% Reb A, and control. Results showed significant increases in caries and bacterial counts only in the sucrose group, while STE and Reb A did not cause dental caries compared to controls. A key limitation was the use of non-human subjects (74). In periodontal disease models with *Porphyromonas gingivalis* infection, 0.1% STE reduced alveolar bone resorption, osteoclast activity, pro-inflammatory cytokines (IL-6, TNF-α, IL-1β), bone loss, and bacterial invasion compared to a 10% glucose control. *In vitro* studies showed that 0.1% w/w STE inhibited *Porphyromonas gingivalis* activity, biofilm formation, and virulence gene expression in a dose-dependent manner (19). A study examined the antimicrobial activity of stevia leaf extracts against cariogenic bacteria and their effects on enamel demineralization compared to glucose and fructose. Seventy-two premolars were divided into six groups: 20% acetone, ethanol, methanol, and water extracts of stevia, and 20% glucose and fructose solutions. After 28 days of culture in a cariogenic substance, the results showed that methanol and water extracts reduced caries depth, while ethanol and acetone extracts had no effect on caries. Additionally, stevia extracts caused less enamel demineralization than glucose and fructose (75).

Gamboa and Chaves tested stevia extract obtained through hexane, methanol, ethanol, ethyl acetate, and chloroform extractions on 16 strains of Gram-positive bacteria (including *Streptococcus mutans, Streptococcus sobrinus*, and *Lactobacillus acidophilus*) and lactic acid bacteria. All extracts showed antimicrobial activity with minimum inhibitory concentration (MIC) range of 30–120 mg/mL. Ethanol and methanol extracts had identical MIC values (120 mg/mL), while the hexane extract had a lower MIC (30 mg/mL). Ethyl acetate and chloroform extracts showed broader inhibition zones at 60 mg/mL (76). These findings highlight the potential of stevia extract as antimicrobial agents; however, their efficacy against dental caries pathogens remains underexplored. Further studies, including one by Guo et al., showed that STE significantly inhibited the growth and acid production of *Streptococcus mutans*, altered biofilm structure, and reduced biofilm viability and extracellular polysaccharide production. STE also reduced the morphological transformation and pathogenicity of *Candida albicans* and was effective in mitigating its virulence factors compared to xylitol. This study is the first to confirm STE’s anticariogenic effects in a dual-species biofilm model, highlighting its potential as a sucrose substitute for reducing dental caries risk (18).

Future clinical trials should be designed to rigorously assess the anti-caries efficacy of stevia across diverse populations, with a particular focus on pediatric cohorts. These trials would provide valuable insights into the potential of stevia as a natural alternative to sucrose in dental care products such as toothpaste and mouthwash. Long-term studies are essential to evaluate the sustained safety and effectiveness of stevia in preventing dental caries and maintaining overall oral health. Additionally, research should extend to examining the broader impact of stevia on the oral microbiome, including its effects on various oral pathogens and the overall balance of the oral microbial community.

### 5.7 Anti-tumor

Over the past two decades, substantial progress has been made in researching the anti-cancer effects of stevia sugar and its metabolites. Numerous studies have elucidated the anti-cancer mechanisms and effects of stevia sugar and its various metabolites.

Yasukawa et al. found that a mixture of STE (48.9%), Reb A (24.4%), Reb C (9.8%), dulcoside A (5.6%), and unidentified components (11.3%) significantly inhibited mouse skin tumor formation induced by 7,12-dimethylbenz[a]anthracene (DMBA) and promoted by the tumor promoter TPA (77). Akihisa et al. discovered that STE, isosteviol, and its five metabolites (7β-hydroxyisosteviol, 7-oxoisosteviol, 11β-hydroxyisosteviol, 12β-hydroxyisosteviol, and 17-hydroxyisosteviol) inhibited the activation of Epstein-Barr virus early antigen (EBV-EA) in Raji lymphoma cells induced by TPA (78). In addition, a large number of *in vitro* experiments have shown that SGs inhibit various cancers, including gastric cancer, colorectal cancer, pancreatic cancer, hepatocellular carcinoma, bladder cancer, ovarian cancer, and breast cancer (20, 78–81). This may be due to SGs’ ability to enhance the sensitivity of cancer cells to anticancer drugs, such as the thymidylate synthase inhibitor 5-fluorouraci (78). At the molecular level, stevia components (such as isosteviol, IC50 = 64 μM) interfere with the activity of DNA topoisomerase II and DNA polymerase, disrupting the DNA replication and repair processes of tumor cells (82).

The anticancer effects of stevia’s active ingredients demonstrate a variety of molecular mechanisms. A *in vitro* study has shown that STE selectively inhibits bladder cancer cell viability and induces mitochondrial stress and apoptosis, with no significant toxicity to normal cells (20). In-depth mechanistic studies indicate that STE activates the GSK-3β signaling pathway by promoting ROS accumulation. On the one hand, it facilitates the degradation of the anti-apoptotic protein Myeloid cell leukemia sequence 1 mediated by F-box and WD repeat domain-containing protein 7, while on the other hand, it triggers endoplasmic reticulum stress to upregulate the expression of apoptosis-inducing protein Phorbol-12-myristate-13-acetate-induced Protein 1 (Noxa) (20). This synergistically activates Bcl-2-associated X Protein (Bax), ultimately leading to cancer cell apoptosis (20). This mitochondrial pathway-mediated apoptosis mechanism is also present in Steviol’s effect on gastric cancer cells. It demonstrates a dose-dependent inhibition of proliferation in various gastric cancer cells (Caco-2, HCT-8, HCT-116, MKN-45, MGC-803, HGC-27), and at concentrations of 100–200 μg/mL, it is comparable to the inhibition caused by 5-fluorouracil, accompanied by an increased Bax/B-cell Lymphoma-2 (Bcl-2) ratio and the activation of cell cycle arrest proteins p21 and tumor suppressor gene p. 53 (79). Notably, Steviol’s (IC50 = 185 μM) effect on breast cancer cells shows a more complex regulatory pattern. Research indicates that STE dose-dependently induces cell cycle arrest at G1 and G2/M phases in breast cancer MCF7 cells, suggesting its multi-target intervention in cell cycle progression (80). Interestingly, SGs demonstrate differential regulatory effects on breast cancer cells under varying endoplasmic reticulum stress conditions. While they induce apoptosis and reduce Bcl-2 expression in both MCF-7 (10, 25, and 40 μM) and MDA-MB-231 (100, 250, and 400 μM) cells, treatment with SGs enhances cell migration and adhesion in low-metastatic ERα + MCF-7 cells, whereas in highly metastatic ERα-/ERβ + MDA-MB-231 cells, SGs significantly inhibit cell migration and metastatic potential (81). This finding suggests that stevia’s active ingredients might exert specific regulation on breast cancer cells of different molecular subtypes through mechanisms such as epigenetic reprograming.

Current research on stevia components’ anti-cancer potential is mainly based on *in vitro* experiments and animal models, providing a crucial foundation for developing novel anti-cancer formulations. The multi-target action of steviol and its derivatives shows promise in cancer therapy. Future research could focus on: (1) Optimizing the structure of stevia’s active components using computer-aided drug design to improve targeting and bioavailability. (2) Developing tumor microenvironment-responsive delivery systems, such as nanoparticles for targeted release at tumor sites. (3) Exploring synergistic effects with conventional chemotherapy to reduce side effects and counteract drug resistance. (4) Using humanized tumor organoid models to assess the inhibitory effects of stevia components on various tumor subtypes. These directions could fully unlock stevia’s therapeutic potential in cancer treatment, offering hope for patients and advancing oncology.

### 5.8 Anti-diarrhea

The potential application of SGs in treating diarrhea was initially derived from observations of the bactericidal and anti-rotavirus activities of stevia extract. Tomita et al. first reported that the aqueous extract of stevia exhibited bactericidal effects against various food-borne pathogens, including enterohemorrhagic *Escherichia coli*, which is known to cause severe hemorrhagic diarrhea. This discovery opened up a new avenue for exploring the application of SGs in diarrhea treatment (83). Subsequently, Takahashi et al. demonstrated that stevia fermented aqueous extract ≥ 10 or 40% (v/v) could inhibit rotavirus growth by interfering with its binding to host cells (84). As an RNA virus, rotavirus is likely to cause childhood gastroenteritis upon infecting humans. In-depth research revealed that the stevia extract might bind to 37 kDa VP7 (Rotavirus protein) and use steric hindrance to interfere with the binding of VP7 to cell receptors, thereby blocking the virus’s attachment to cells and exerting an anti-diarrhea effect (84).

Shiozaki et al. pointed out that stimulating the contraction of intestinal smooth muscle could cause significant hypermotility diarrhea, whereas the aqueous extract of stevia could inhibit this contraction (85). Further research showed that when the concentration of STE was 1 mM, it could inhibit the contraction of isolated guinea-pig ileum induced by CaCl*2* (10 mM) by 40%. Analysis revealed that this mechanism was closely related to its inhibitory effect on the influx of Ca^2+^ into muscle cells (21). The intake amount and frequency of stevia may affect changes in the colon microenvironment and have potential benefits for the α-diversity of the microbiome (86). Stevia extract may improve gut health and anti-diarrheal effects by enhancing the villus height/crypt depth ratio in the duodenum, promoting microbial α-diversity in the cecum, and significantly altering the abundance of *Bacteroidetes* and *Firmicutes*, with a suggested effective inclusion level of 200 mg⋅kg^–1^ in poultry feed (87). Nowadays, STE and Reb A have been added to the diet of piglets to reduce the incidence of diarrhea (88).

Based on the above research findings, it can be clearly concluded that stevia sugar has potential value in treating diarrhea caused by excessive intestinal motility, such as irritable bowel syndrome and inflammatory bowel disease. However, to comprehensively evaluate the actual therapeutic efficacy of SGs in treating various types of diarrhea, more *in vivo* experiments are still needed, especially studies in animal models for diarrhea caused by different etiologies.

### 5.9 Protect the liver and kidneys

Numerous studies have demonstrated that stevia not only reduces liver and kidney damage in streptozotocin (STZ)-induced diabetic rats but also exerts positive effects on liver injury, liver fibrosis, kidney injury, and other conditions caused by various factors.

In terms of liver protection, stevia leaves (500 mg⋅kg^–1^ oral administration) and STE (250 mg⋅kg^–1^ oral administration) can alleviate structural changes and apoptosis of hepatocytes in rats with LPS-induced acute liver injury, and restore the levels of aspartate aminotransferase (AST) and alanine aminotransferase (ALT) in serum and tissues (89). To enhance bioavailability, functionality, and stability, some researchers prepared stevia loaded in nano-niosomes (nano-stevia) and investigated its protective effect on liver injury in STZ-induced diabetic rats. This study provided evidence that stevia and nano-stevia have anti-diabetic and hepatoprotective effects by targeting the hepatic GLUT-2/GLUT-9 (90). The aqueous extract of stevia (100 mg⋅kg^–1^ by gavage daily) can induce the expression of Nrf2, reduce the expression of NF-κB, block several profibrotic signaling pathways, thereby inhibiting the activation of hepatic stellate cells and preventing chronic carbon tetrachloride (CCl_4_)-induced liver cirrhosis (91). In the db/db mouse model, PPARα-dependent lipophagy is involved in hepatic steatosis. Stevia (200 and 500 mg⋅kg^–1^⋅day^–1^) and STE (40 mg⋅kg^–1^⋅day^–1^) can improve hepatic steatosis by increasing the levels of fatty acid oxidase, PPARα, and microtubule-associated protein light chain 3b in the liver of db/db mice, and reducing the level of sequestosome 1 (p. 62) (12). And *in vitro* study demonstrated that STE, as a PPARα agonist, promotes PPARα -dependent lipophagy, thereby alleviating steatosis in HepG2 cells (12).

Regarding kidney protection, compared with the untreated diabetic group, two doses of the ethanolic extract of the bitter fraction of stevia (200 and 400 μg⋅kg^–1^ body weight) can significantly prevent glomerular hypertrophy and the reduction in the number of glomeruli. A 3-month study on stevia combined with conventional antihypertensive drugs (angiotensin-II receptor blockers) and antidiabetic drugs (Ca^2^ channel blockers) showed that STE can significantly improve serum uric acid and microalbumin in patients with chronic kidney disease (CKD) (92). Another experiment exploring the renoprotective effect of stevia on gentamicin-induced nephrotoxic rats demonstrated that stevia (200 mg⋅kg^–1^⋅day^–1^) can significantly reduce the levels of serum creatinine, liver enzymes, and total serum bilirubin, and mildly alleviate renal tissue damage, inflammation, and tubular necrosis (93). The whole leaf powder of stevia (4% w/w) and the extracted polyphenols can reduce the levels of ALT and AST, as well as the concentration of MDA in the liver, and increase the glomerular filtration rate (94). The stevia residue extract has a protective effect on the kidneys of mice with adenine-induced CKD, improving the histopathology and ultrastructure of the kidney tissues. In addition, stevia residue extract (200 and 400 mg⋅kg^–1^) also alleviates renal fibrosis by inhibiting the TGF-β1/Smad and Wnt/β-catenin signaling pathways and improving the composition of the gut microbiota (95). Stevia aqueous extract (400 mg⋅kg^–1^) exerts nephroprotective effects by regulating the expression of AQP2 mRNA and the activity of the antioxidant signaling pathway Nrf2/Kelch-like ECH-associated protein 1 in the kidneys (11).

To maximize stevia’s therapeutic potential, research should explore the synergistic effects of stevia when combined with existing anti-diabetic or anti-hypertensive medications, aiming to enhance efficacy and minimize adverse effects. Additionally, targeted studies should assess stevia’s efficacy in specific populations, such as children, the elderly, or individuals with a genetic predisposition to liver or kidney diseases, to identify unique benefits or risks. Furthermore, evaluating the impact of stevia on the liver and kidney health of patients undergoing chemotherapy or other drug treatments that may induce organ toxicity could provide critical insights into its role as a supportive therapeutic agent.

### 5.10 Methodological limitations and future directions in *S. rebaudiana* pharmacological effect study

Current research on stevia encompasses *in vitro*, animal (*in vivo*), and human studies, utilizing a wide array of models and investigating diverse pharmacological endpoints. The diversity of models—ranging from cell lines and Caenorhabditis elegans to rodents, pigs, and humans—and the breadth of outcomes studied, including antioxidant, anti-inflammatory, and metabolic effects, collectively suggest broad biological activity. However, significant heterogeneity in study design, methodology, and reporting precludes clear, unified conclusions and limits the strength of the overall evidence base.

Evaluating by study type reveals distinct methodological challenges. In animal studies, a critical limitation is the pervasive use of small sample sizes (typically *n* = 6 to *n* = 10 per group), which reduces statistical power and diminishes the reliability of the findings. The risk of bias in these studies is high, largely due to frequent omissions in reporting randomization, blinding procedures, sample size calculations, and detailed inclusion/exclusion criteria. Furthermore, while study durations vary from acute (4 days) to sub-chronic (up to 15 weeks), there is a notable gap in long-term investigations (e.g., > 6 months). The extreme variation in administered dosages, often without clear translation to human-relevant exposure levels, further complicates the interpretation and extrapolation of results. Human studies, while generally featuring larger sample sizes (e.g., *n* = 15–174), are also hampered by methodological shortcomings. A lack of detailed reporting on randomization, blinding, placebo control, and compliance monitoring significantly increases the risk of bias, undermining confidence that observed effects are attributable solely to stevia intervention. Although the intervention durations in human trials are a relative strength (ranging from 8 weeks to 2 years), inconsistent reporting of dosages (e.g., “1 cup of 2% extract,” “capsules containing 250 mg”) and a near-total absence of systematic dose-response studies prevent the establishment of optimal, clinically relevant intake levels.

Several critical contradictions and shortcomings emerge from this analysis. The most significant limitation is the absence of large-scale, long-term, randomized, double-blind, placebo-controlled trials investigating stevia’s effects on definitive, hard metabolic endpoints. The current literature fails to adequately address whether observed bioactivities translate into long-term clinical benefits, such as the prevention of T2DM, reduction of cardiovascular risk, or improvement of renal function in at-risk populations. Furthermore, findings on core metabolic effects are inconsistent; some studies report lipid improvements without concurrent changes in blood glucose or insulin resistance, while others demonstrate linked benefits. A notable discrepancy exists in human data where reductions in fasting blood glucose are not always reflected in changes in HbA1c, highlighting a gap between acute and chronic glycemic effects. In anti-tumor research, which remains predominantly *in vitro*, one study even noted a context-dependent “dual role” where SGs may under certain conditions promote cancer cell migration, underscoring the complexity and need for cautious *in vivo* validation (81). Finally, translating promising mechanistic insights—largely derived from animal or cellular models—to human physiology remains a major challenge, compounded by the use of varied and poorly characterized stevia preparations across studies, which hinders the identification of specific active components.

In conclusion, while the existing body of research on stevia offers valuable and promising hypothesis-generating evidence, the overall quality of the evidence remains low to moderate. This is primarily due to pervasive issues with small sample sizes, high risk of bias, and a lack of standardized, long-term experimental protocols. The foremost obstacle to translating these preclinical and preliminary clinical findings into evidence-based practice is the scarcity of high-quality, long-term human RCTs. Future research must therefore prioritize large, rigorously designed, multi-center trials that employ standardized stevia formulations and clinically relevant dosages. Such studies are essential to robustly assess long-term metabolic, cardiovascular, and renal outcomes, thereby providing a definitive evaluation of stevia’s therapeutic potential and safety profile for human health.

## 6 Safety

The key findings from the toxicology studies are summarized in Table 3.

**TABLE 3:** Toxicological assessment of *S. rebaudiana.*

Type of toxicological experiment	Study type	Number of samples/test systems	Doses of stevia	Results	References
	*In vitro* (bacteria)	Multiple strains in reverse mutation, forward mutation, *umu* test, *rec* assay	STE (purity: 83.2%) and steviol (purity: 99%). Dose levels: 50, 100, 500, 1,000, and 5,000 μg per plate	Negative in all bacterial assays	(96)
	*In vitro* (mammalian cells)	Chinese hamster lung fibroblast cell line (chromosomal aberration test, gene mutation assay)		Negative in both assays.	
	*In vivo* (mice)	Mouse micronucleus test		Negative.	
	*In vitro (bacteria)*	*Salmonella typhimurium* TM677 (forward mutation)		Positive (dose-related, requires S9 metabolic activation).	
		*Salmonella typhimurium* TA1535/pSK1002 (*umu* test)		Weakly positive (with or without S9).	
		*Salmonella typhimurium* TA97, TA98, etc., & *E. coli* WP2 (*reverse mutation*)			
		*B. subtilis* (*rec* assay)		Negative (even with S9).	
	*In vitro* (mammalian cells)	CHL cells (chromosomal aberration, gene mutation assay)		Positive (dose-related, requires S9 metabolic activation).	
	*In vivo* (mice)	Mouse micronucleus test		Negative.	
	*In vitro* (human lymphocytes)	Whole-blood from 5 healthy donors	STE: 1, 5, 10 mg/mL Steviol: 0.1, 0.2 mg/mL	Results from this specific experiment are not provided in the given text (the text states these concentrations were tested).	(97)
	Ames Test (bacterial reverse mutation)	Triplicate plates per dose, repeated twice	62, 185, 556, 1,667, 5,000 μg/plate	Negative: No significant increase in revertant colonies, with or without metabolic activation (S9).	
	Mouse bone marrow micronucleus assay	5 Males + 5 females per group, total 50 mice	2,500, 5,000, 10,000 mg/kg BW (oral gavage, twice)	Negative: No significant increase in micronucleated polychromatic erythrocytes; no evidence of chromosomal damage.	(98)
	Mouse sperm malformation assay	7 Male mice per group, total 35 mice	2,500, 5,000, 10,000 mg/kg BW (oral gavage, daily for 5 days)	Negative: No significant increase in sperm malformation rates.	
Carcinogenic potential studies	*In vivo*	45 Males + 45 females per group (4 groups)	0, 0.2, 0.6, 1.2% in diet for 2 years	No treatment-related toxicity in: body weight food consumption mortality hematology clinical biochemistry organ weights Histopathology Tumor incidence Maximum NOEL = 1.2% in diet ADI = 7.938 mg/kg bw/day (humans)	(23)
	*In vivo*	50 males + 50 females per group (3 groups)	0, 2.5, 5% (95.6% pure STE)	No carcinogenic effect observed. No significant increase in tumor incidence in any organ/tissue. Reduced mammary adenoma incidence in females (linked to reduced body weight gain). Reduced severity of chronic nephropathy in males. Lower final survival in 5% male group due to spontaneous LGL leukemia (not treatment-related). STE is not carcinogenic in F344 rats.	
	*In vivo* (rat)	50–90 Rats/sex/group (4 studies)	0.1–1% Stevia extract 0.2–1.2% STE 2.5–5% STE	No treatment-related increase in tumors or non-neoplastic lesions. Reduced mammary adenomas in female F344 rats. No carcinogenic effects observed.	(99)
	*In vivo* (mouse)	Transgenic C57BL/6 Ela1-Tag mice	0.02% w/v stevia in drinking water (*in utero* to 21 weeks)	No effect on pancreatic acinar carcinoma development, growth, or mortality	
Acute and subchronic oral toxicity studies	*In vivo*	40 BALB/c mice (20 males, 20 females)	470, 620, 940, 1,880 mg/kg (oral, 4 weeks)	Increased oxidative damage (↑TOS, ↓TAS, ↓PON-1 activity, ↑OSI). Increased chromosomal aberrations (↑% abnormal cells, omal aberrations (↑) doses (940 and 1,880 mg/kg). Increased mitotic index (↑cell division activity). No significant effect on HDL-C/LDL-C levels.	(100)
	*In vivo*	10 Males and 10 females per group (total 6 groups)	0, 0.31, 0.62, 1.25, 2.5, and 5% STE in diet	No mortality during the 13-week administration period. No differences in body weight gain or food consumption between control and treated groups. Increased lactate dehydrogenase levels and single cell necrosis in the liver of all male treated groups, but these changes were not considered specific due to lack of clear dose response, low severity, and limitation to males. Other hematological and biochemical differences were of minor toxicological significance. A concentration of 5% in diet was concluded to be a suitable maximum tolerable dose for a 2-year carcinogenicity study in rats	(101)
Genotoxicity studies	*In vitro* (human peripheral blood lymphocytes)	Two repetitive experiments using whole-blood cultures	Negative control (pure water), 1, 2, 4, 8, and 16 μg/mL (equivalent to ADI/4, ADI/2, ADI, ADI × 2, ADI × 4)	No significant difference in chromosomal aberrations or micronuclei induction between stevia-treated groups and negative control at 24 and 48 h. Stevia showed no genotoxic activity in both test systems.	(102)
	*In vitro*	Human lymphocytes exposed to stevia	Stevia: 5%, 0.5%, 0.05% for 2 h	No significant genotoxic activity; possesses antigenotoxic activity at all tested concentrations	(103)

### 6.1 Mutagenicity studies

Studies show that both STE and steviol do not exhibit mutagenic effects. In tests involving *Salmonella typhimurium* TA98 and TA100, as well as cultured human lymphocytes, no mutagenicity was observed for STE. For steviol, no mutagenic effects were seen at concentrations up to 25 mg per plate, while 50 mg per plate showed mutagenicity only toward TA98. Additionally, experiments with blood lymphocytes cultured from healthy donors revealed no chromosomal effects (96, 97). In a bacterial reverse mutation assay, *Salmonella. typhimurium* strains TA97, TA98, TA100, and TA102 were exposed to up to 5,000 μg/plate of stevia extract, both with and without metabolic activation, with no mutagenic effects observed (98).

### 6.2 Carcinogenic potential studies

A 108-week study on F344 rats showed that SGs did not exhibit carcinogenic effects at doses of 0, 2.5, and 5%. Similarly, a 2-year study on Wistar rats at doses of 0, 0.2, 0.6, and 1.2% SGs found no significant changes in growth, feed consumption, mortality rate, or lifespan, with no carcinogenic lesions observed. Further genotoxicity testing confirmed that SGs showed no mutagenicity or carcinogenic activity (23, 24). A comprehensive review of over 900 carcinogen-related endpoints also found no evidence of carcinogenicity in SGs (99).

### 6.3 Acute and subchronic oral toxicity studies

In a 4-week study, BALB/c mice (both male and female) were administered SGs at doses of 470, 620, 940, and 1,880 mg⋅kg^–1^. The findings revealed that SGs slightly increased oxidative damage, cell cycle activity, and the frequency of chromosomal aberrations (100). Additionally, no hepatotoxicity, Ames toxicity, or skin sensitivity were observed in rats exposed to stevia extract (39). A 13-week subchronic study in F344 rats with dietary doses of 0–5% SGs showed no mortality, significant weight changes, or major health effects, though mild liver necrosis was observed in males, which was non-specific and not dose-dependent (101).

### 6.4 Genotoxicity studies

*In vitro* and *in vivo* studies showed no genotoxic effects from SGs or steviol at various concentrations. While a high-dose (1,880 mg⋅kg^–1^) study in mice showed slight increases in oxidative damage and chromosomal aberrations, no genotoxic effects were found at doses up to 8,000 mg⋅kg^–1^ in other studies (21, 100). The concentrations of stevia (active component STE) at ADI/4, ADI/2, ADI, ADI × 2 and ADI × 4 added to whole blood culture showed no genotoxic activity in human peripheral blood lymphocytes during the 24 and 48 h treatment periods (102). It is worth noting that 0.5% w/w and stevia not only did not cause significant genetic damage, but also exhibited antigenotoxic activity at concentrations of 5, 0.5, and 0.05% (103). Given the extremely high doses used in these experiments, it is necessary to determine the therapeutic index of SGs for healthy volunteers to clarify the dosage range for their potential pharmacological applications.

### 6.5 Critical summary of the toxicological studies of *S. rebaudiana*

Based on comprehensive toxicological data, the safety profile of SGs, particularly STE, is well-supported by a robust body of evidence, although the quality of individual studies varies. The evidence base is primarily comprised of high-quality, standardized *in vivo* animal studies with adequate sample sizes (e.g., carcinogenicity studies involving 45–50 animals per sex per group) (23, 24, 99). These studies consistently show a clear absence of carcinogenic, teratogenic, or acute toxic effects at relevant dietary exposures, leading to the establishment of a well-defined ADI.

However, this strong foundation is complicated by contradictory *in vitro* genotoxicity findings. While the vast majority of assays, including rigorous Ames tests and *in vivo* micronucleus assays, consistently yield negative results, isolated positive findings for the metabolite steviol are reported in certain sensitive test systems (e.g., forward mutation in *Salmonella typhimurium* TM677 and chromosomal aberrations in CHL cells), but only under metabolic activation (S9 mix) (96). This discrepancy between negative *in vivo* outcomes and positive *in vitro* signals under artificial conditions represents a significant gap, likely due to species-specific metabolic differences and the high concentrations used in *in vitro* studies, which are not achievable through oral consumption.

Furthermore, a significant research gap exists in the lack of long-term, prospective human studies. While historical consumption in certain populations provides anecdotal reassurance regarding safety, there is a notable absence of large-scale, randomized controlled trials designed to monitor long-term genotoxic, metabolic, or microbiome endpoints in diverse human populations consuming SGs at levels near the ADI over extended periods.

### 6.6 Safe intake amount of *S. rebaudiana*

The global regulatory landscape for SGs is anchored by a robust safety assessment, leading to harmonized intake limits. The Joint FAO/WHO Expert Committee on Food Additives (JECFA) recommends a safe ADI of 4 mg⋅kg^–1^ body weight of SGs equivalent, which is about 12 mg of high-purity stevia extract per kg of body weight (104). In alignment with this, the EFSA Panel on Food Additives has extended its approval, recognizing that the existing toxicological database supports the safety of a broader range of SGs. Specifically, EFSA amended the specifications to include up to 60 SGs derived from *Stevia rebaudiana* leaves (maintaining a 95% purity threshold), confirming that the same ADI of 4 mg⋅kg^–1^ bw/day applies to the entire group, thereby facilitating innovation within defined safety parameters (25). FDA operates through the GARS notification process, through which specific, high-purity SG preparations (including various Rebs like Reb A and Reb M) have been approved for use as general-purpose sweeteners (105, 106).

The advent of fermentation-derived SGs, produced using engineered microbes like *Yarrowia lipolytica*, introduces important nuances into this regulatory framework. EFSA has evaluated these novel production methods on a case-by-case basis, concluding that the final, purified products are toxicologically equivalent to their plant-derived counterparts, and thus the existing ADI remains applicable (25, 107). However, EFSA stipulates that separate specifications are required for fermentation-derived additives due to potential differences in impurity profiles, ensuring safety assessments address production-specific compounds (107, 108). This has direct implications for labeling. In jurisdictions like the EU and the U.S., ingredients must be declared by their common or usual name. While the resulting molecules are chemically identical, labeling may need to reflect the production method (e.g., “SGs from fermentation” or “enzyme-modified SGs”) to inform consumers and ensure regulatory transparency, even though the safety standard (the ADI) is the same (26).

### 6.7 Interactions between *S. rebaudiana* and the gut microbiome

Based on a critical synthesis of the provided evidence, the interaction between stevia consumption and the gut microbiome is subtle and dose-dependent, with no indication of significant dysbiosis at typical human intake levels but with modulatory effects that preclude definitive causal claims. High-quality human intervention studies indicate a reassuring safety profile. High-quality human intervention studies report a reassuring safety profile. A 12-week trial in healthy adults (*n* = 14 per group) consuming stevia drops (five drops, twice daily), found no significant alteration in overall microbial diversity (alpha/beta diversity) or major taxa abundance (109). This aligns with data showing that intake within the recommended ADI—including continuous consumption of 25% of the ADI for 4 weeks (*n* = 36/23)—does not adversely affect gut microbiota, short-chain fatty acid profiles, or cardiometabolic indicators (27). *In vitro* studies using fecal inocula further support this, demonstrating that Reb D, Reb M, and steviol at 200 μg/mL do not alter bacterial population structure in samples from men, women, or children (*n* = 6 per group) (110). However, sensitive analytical methods (e.g., random forest analysis) can still distinguish subtle, community-wide compositional shifts between consumers and controls, though their clinical relevance remains unclear (109).

In animal models, evidence suggests greater potential for microbial modulation. A study in healthy rats showed that 9 weeks of exposure to 10% (w/w) Reb A in drinking water (*n* = 8/group) altered specific microbial taxa without affecting host weight or glucose tolerance (111). Research in metabolically dysregulated models indicates a more pronounced effect. For instance, maternal STE supplementation (0.5 mg/mL in drinking water during gestation and lactation; *n* = 10/group) in obese mouse dams improved offspring metabolic outcomes, an effect mediated by increased abundance of specific bacteria like *Lactobacillus apodemi* (112). This indicates that stevia’s microbial impact may be context-dependent, varying with host metabolic status, dose, and duration (86). Notably, the metabolism of SGs itself is gut microbiota-dependent, which establishes a fundamental, bidirectional interaction (86).

A critical limitation across the field is the scarcity of long-term, longitudinal human data. Existing human trials are largely short-term (≤ 12 weeks) and conducted in healthy populations. As noted in reviews, alterations in the gut environment may depend on the amount, frequency of intake, and concomitant diet (86). To move beyond correlation and establish causality, prospective, longitudinal human studies combining detailed microbial multi-omics with robust clinical phenotyping are urgently needed, especially in vulnerable populations. Current evidence, while supporting safety within the established ADI of 4 mg⋅kg^–1^ bw/day, is insufficient to conclusively define stevia as a prebiotic or to predict its chronic, population-wide effects on gut ecosystem stability. Epidemiological data further reinforce the safety profile, showing no consistent correlation between SG consumption and allergic diseases (113).

### 6.8 Unanswered safety questions

Despite a strong scientific consensus on the safety of SGs, a rigorous and critical examination reveals several areas that warrant further investigation to fully complete the risk assessment profile:

First, the mechanisms underlying high-dose effects observed in animal studies—such as oxidative stress and chromosomal aberrations at extreme exposure levels (100), while not directly relevant to human exposure, warrant a mechanistic investigation. Is this a species-specific effect? Does it represent a threshold-based overwhelm of metabolic pathways? Understanding this is key to definitive risk characterization.

Second, while the safety of SGs for the general population is well-supported, there remains a scarcity of long-term, low-dose exposure data for vulnerable subpopulations. This includes pregnant women, infants, and individuals with impaired gut barrier function, such as those with inflammatory bowel disease. Future research should focus on human biomonitoring studies and advanced toxicokinetic modeling to resolve the *in vitro/in vivo* discrepancies for steviol and to confirm the long-term safety of SGs in varied dietary contexts. Additionally, specific research gaps persist, such as the metabolic patterns of stevia in livestock and poultry. The possibility of subtle yet persistent modulation of the gut microbiome in these groups warrants targeted and longitudinal study.

Third, the potential for drug-transporter interactions represents a relatively unexplored area of research. Specifically, the capacity of steviol—the common aglycone metabolite of all SGs—to interact with human renal transporters remains understudied. The unclear mechanism of SGs’ effect on renal transport proteins also requires clarification. Although no clinical evidence currently suggests such interactions alter drug pharmacokinetics, this theoretical possibility merits systematic evaluation.

Fourth, while the toxicological focus has rightly centered on steviol, limited attention has been paid to minor bacterial metabolites or processing-related compounds. For instance, the safety implications of thermal degradation products generated during high-temperature processing or baking, as well as trace metabolites derived from microbial transformation, have not been fully elucidated.

Fifth, it is important to note that safety evaluations and Generally Recognized as Safe (GRAS) determinations are substance- and process-specific. Therefore, not all rebaudioside products or novel production methods (e.g., those using new fermentation strains) are automatically covered by existing data, and each requires its own independent safety evaluation.

Finally, an important non-scientific but highly relevant issue concerns the public perception of safety based on the “natural” origin of SGs. It is essential to clearly communicate that safety is determined solely by toxicological evidence—not source—and that the ADI applies equally to both plant-derived and biosynthetically produced (fermented) SGs, provided they are chemically identical.

## 7 Application

The use of stevia has evolved significantly, transforming from a specialized natural sweetener into a versatile bio-resource with a wide range of applications across the food, health, agricultural, and even environmental sectors. This shift is driven by an increasing recognition of its functional properties beyond just sweetness, positioning it as a key contributor to the development of sustainable and health-promoting products.

### 7.1 Food and beverage industry

The food and beverage industry remains the primary and most established sector for SGs, where it serves as a widely accepted sucrose substitute. Following key regulatory approvals, such as the Food and Drug Administration’s (FDA) GRAS designation and EFSA’s authorization, SGs has been incorporated into a broad range of products, including beverages, dairy items, confectionery, and baked goods. It is often blended with other sweeteners, such as erythritol or allulose, to balance taste, reduce costs, and compensate for the loss of bulking properties provided by sugar (114, 115). However, challenges persist, particularly the bitterness and licorice-like aftertaste associated with certain glycosides like STE. To address these challenges, several strategies are employed: the preferential use of high-purity Reb A for its cleaner taste profile; advanced flavor masking and encapsulation techniques utilizing carriers such as inulin or maltodextrin (116); and a growing shift toward next-generation glycosides like Reb M and Reb D, which offer a taste profile closer to sucrose and reduce the need for extensive masking in premium formulations (117).

### 7.2 Functional foods and nutraceuticals

Beyond its role as a natural sweetener, stevia is increasingly recognized as a key functional ingredient in the health and wellness sector, where its bioactivities complement its sweetening properties to create versatile product offerings. In the context of gut health, incorporating SGs into dairy products or supplements not only reduces caloric content but also exerts a prebiotic-like effect by selectively promoting beneficial bacteria such as *Bifidobacteria* and *Lactobacilli*, thereby supporting a balanced gut microbiota (118). In the realm of metabolic health, stevia’s anti-hyperglycemic and anti-hyperlipidemic properties are leveraged in tablets, oral liquids, and functional foods designed for diabetics and individuals with metabolic syndrome. These products offer not only sweetness but also potential benefits for blood glucose management and weight control, transforming stevia from a passive sugar substitute to an active nutritional intervention (118). Additionally, in oral care applications, Herbal mouthwash formulated with neem and stevia extracts exhibits compelling antimicrobial effects against key oral pathogens such as *Staphylococcus aureus*, *Enterococcus faecalis*, and *Candida albicans*, suggesting its potential as an effective alternative for long-term use (119).

### 7.3 Animal feed industry

A promising and high-value application involves using stevia-derived compounds—specifically chlorogenic acid and isochlorogenic acid extracted from stevia leaf residue—as functional feed additives in animal production. In poultry, dietary supplementation with stevia chlorogenic acid has been shown to enhance antioxidant capacity and support intestinal health in laying hens (87, 120). Similarly, in pigs, stevia residue extract has demonstrated significant benefits: for weaned piglets, it reduces the incidence of diarrhea, improves antioxidant status, and positively modulates gut microbiota (49), while in finishing pigs, it promotes growth performance, meat quality, and immune function (121). Beyond enhancing animal health and productivity, this application adds substantial value to the stevia processing industry by converting agricultural by-products into valuable functional ingredients.

### 7.4 Environmental remediation

Perhaps the most innovative application of stevia lies in the field of environmental science, where its waste is transformed into a valuable resource for pollution control, embodying the principles of a circular bio-economy. Stevia leaves and their processing residues can be repurposed into highly effective biosorbents for wastewater treatment. For instance, chemically modified stevia residue has demonstrated excellent efficiency, adsorbing up to 92% of toxic cadmium ions (Cd^2+^) from aqueous solutions (122). Similarly, stevia-based adsorbents treated with sodium hydroxide can effectively remove malachite green dye from wastewater (123). Additionally, activated carbon derived from stevia leaves shows strong potential for fluoride removal, aiding in water defluoridation (124). Beyond adsorption, stevia also contributes to advanced oxidation processes: zinc oxide nanoparticles synthesized using stevia leaf extract serve as a green and efficient photocatalytic agent, capable of degrading persistent antibiotic pollutants like ciprofloxacin and tetracycline. These nanoparticles are not only highly effective but also recyclable, offering a sustainable and innovative approach to wastewater purification (125).

### 7.5 Agricultural applications

Returning stevia residue, whether in its fresh or composted form, to the soil represents a sustainable agricultural practice that effectively closes the resource loop. Studies show that this soil amendment, especially when combined with chemical fertilizers, significantly enhances soil quality. Key benefits include increased activity of essential soil enzymes such as dehydrogenase, invertase, and urease, as well as the promotion of beneficial microorganisms like *Bacillus* spp. Ultimately, these improvements lead to higher biomass yields and elevated Reb A content in subsequent crops of stevia (126). This approach not only revitalizes soil health but also reduces reliance on synthetic fertilizers.

## 8 Biosynthesis of next-generation SGs-Reb M

The quest to overcome the sensory limitations of native SGs has spurred advancements in biosynthesis. The primary goal is to efficiently and cost-effectively produce rare, superior-tasting glycosides, particularly Reb M, which offers a clean, sugar-like taste but is found in trace amounts (< 0.5%) in the stevia plant (117). As such, traditional plant extraction methods are commercially and ecologically unfeasible for large-scale Reb M supply. Consequently, this chapter focuses exclusively on the advanced biotechnological routes—enzymatic bioconversion and microbial fermentation—that constitute the field of Reb M biosynthesis.

### 8.1 The shift from extraction to biosynthesis

While direct plant extraction of Reb M is conceptually simple, involving processes like crushing, ethanol immersion, and chromatography (127, 128), it is commercially impractical due to the compound’s extremely low natural abundance, resulting in prohibitively high costs and unsustainable resource demands (117). This fundamental limitation has driven research and development decisively toward synthetic biology approaches. The biosynthesis of Reb M, whether via enzymatic or fermentative routes, represents a more scalable, efficient, and economically viable pathway for industrial production, moving beyond the constraints of agricultural supply chains.

### 8.2 Biosynthetic routes and technical strategies for Reb M production

**FIGURE 5:**
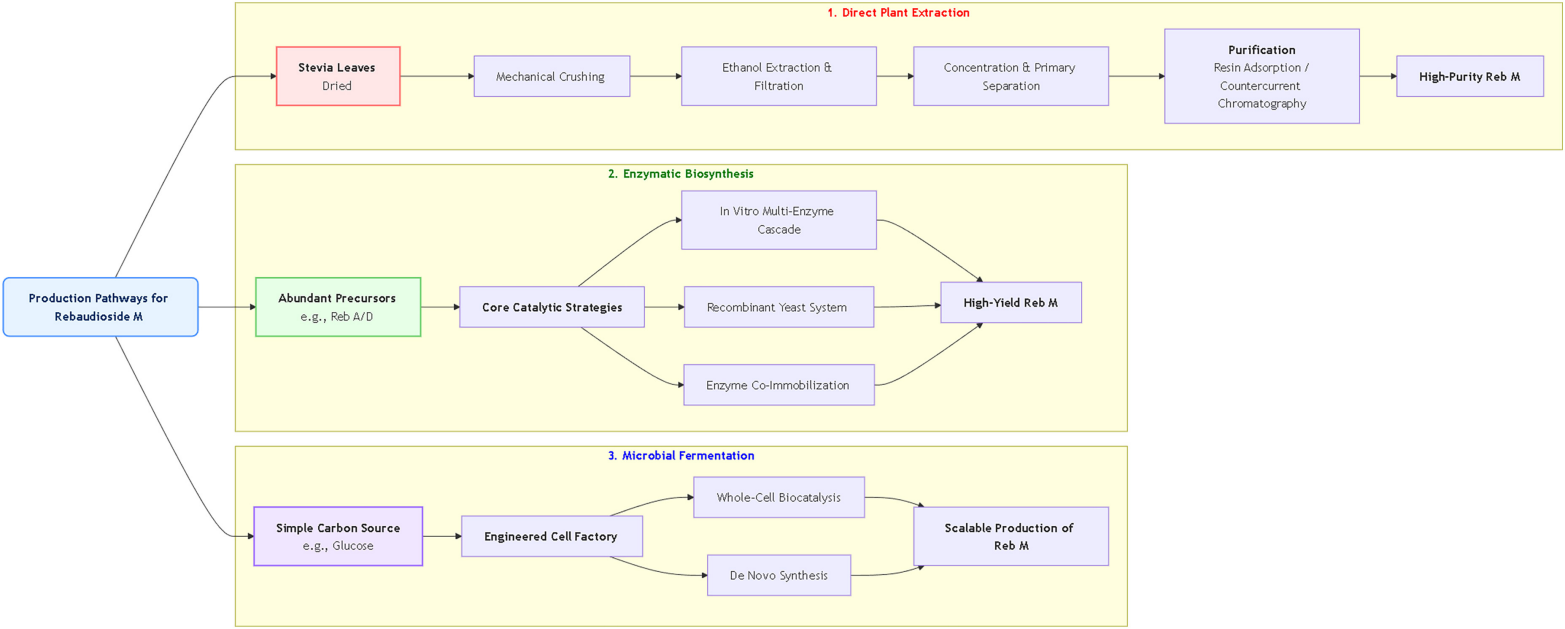
Reb M biosynthesis method flowchart.

The biosynthesis of Reb M can be achieved through two principal, and often complementary, technological strategies. The biosynthesis method of Reb M is shown in Figure 5. For a comparative analysis of the technical characteristics, industrial feasibility, and latest research advancements, refer to Table 4.

**TABLE 4:** Comparison of Reb M production routes: analysis and case.

Production route	Core strategy	Key advantages	Key disadvantages/ dependencies	Regulatory status and notes	Case analysis
Direct plant extraction	Extracting Reb M directly from *Stevia rebaudiana* leaves.	Natural source, simple concept.	Commercially non-viable: Extremely low natural abundance (< 0.5%), high cost, low yield, unstable supply, environmentally intensive (117).	Must comply with general steviol glycoside regulations. Impractical for market supply.	No viable performance data. The low intrinsic yield precludes competitive production.
Enzymatic bioconversion	Using engineered glycosyltransferases to convert abundant precursors (e.g., Reb A, Reb D) into Reb M.	High specificity and purity; mild reaction conditions (150); leverages existing supply chains; high conversion rates achievable (131).	Dependent on precursor (Reb A/Reb D) supply/cost. requires efficient, stable enzyme production.	Classified as “enzyme-modified” SG. gaining recognition (e.g., RebM2 has FDA GRAS) (105).	Case 1: Fusion enzyme (UGT76G1-91C1) increased synthesis rate 1.8-fold (150) case 2: mutant SrUGT76G1 (M88V) achieved 92.4% yieldfrom Reb D (137) case 3: variant UGT76G1 enabled 90.5% yieldat scale (136) case 4: engineered UGT94D1 produced Reb M2 with 92% yield (151).
Microbial fermentation	Engineered microbes (e.g., *Yarrowia lipolytica*) synthesize Reb M *de novo* from simple sugars in bioreactors (26).	Scalable and efficient; independent of plants; high theoretical yield and cost control; suitable for large-scale production.	High initial R&D/capital investment; complex fermentation/purification; requires strict process control.	EFSA concluded fermentation-derived Reb M (using *Yarrowia lipolytica* VRM) is safe; existing ADI (4 mg/kg bw/day) applies, but separate specs are required (107).	Case: Engineered *Saccharomyces cerevisiae* produced 12.5 g/L Reb M (77.9% yield from STE), the highest reported titer (131).

This strategy utilizes isolated or cell-bound enzymes to convert more abundant and cost-effective SG precursors, such as Reb A or STE, into high-value Reb M.

Enzymatic biosynthesis has emerged as the most active and promising production route, primarily involving: *in vitro* multi-enzyme cascade reactions, *Saccharomyces cerevisiae* enzymatic processes, and co-immobilized enzyme technology (129, 130). By utilizing engineered glycosyltransferases (UGTs) to efficiently convert more abundant precursors, such as Reb A or Reb D, into Reb M, significant advancements have been made. These include optimized *in vitro* multi-enzyme cascades that achieve high yields, recombinant Saccharomyces cerevisiae systems for enhanced catalysis, and co-immobilized enzyme technologies that reduce process costs.

**FIGURE 6:**
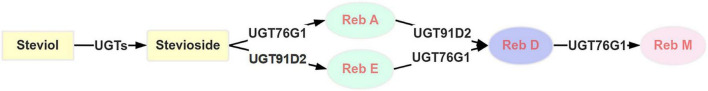
Two UGT76G1-dependent pathways for the biosynthesis of Reb M via microbial fermentation using biomaterials with either constant or variable glucose content.

This more integrated strategy employs engineered microbial cell factories (e.g., *Yarrowia lipolytica, Escherichia coli*) to synthesize Reb M *de novo* from simple carbon sources like glucose, entirely independent of the stevia plant (26, 107, 131). The biosynthetic pathway in microbes mirrors and reconstructs the plant’s natural pathway. All SGs originate from the central diterpenoid pathway, yielding the aglycone steviol. A family of UDP-dependent glycosyltransferases (UGTs) then sequentially add glucose moieties to steviol (132). The microbial pathway to Reb M proceeds through two predominant UGT76G1-dependent routes (illustrated in Figure 6): the first is a stepwise pathway: steviol is initially glycosylated to form STE, which is further glycosylated at the C13 sophorose chain to yield Reb A. Reb A is then converted to Reb D through the addition of a glucose via a β(1–2) linkage. Finally, UGT76G1 acts on Reb D, catalyzing the transfer of a glucose moiety via a critical β(1–3) linkage to form Reb M (26). Alternatively, a branch pathway exists via Reb E: STE can be converted to Reb E, which is subsequently transformed into Reb D, and ultimately glycosylated again by UGT76G1 to produce Reb M (133). The regio-specificity and linkage specificity exhibited by these UGTs—particularly the distinction between β(1–2) and β(1–3) glycosidic bonds—are of paramount importance, as they directly dictate the sensory properties of the final glycoside.

### 8.3 The key catalyst: UGT76G1 and its solubility challenge

UGT76G1 is the key enzyme responsible for the final, value-adding step in the synthesis of Reb M. However, its heterologous expression—especially in the high-yield prokaryotic host *Escherichia coli*, a preferred workhorse for industrial enzyme production—has been severely limited by a major bottleneck: poor solubility and a strong tendency to form inclusion bodies. This has been the primary obstacle to achieving efficient, large-scale production of the enzyme. In response, substantial research efforts have focused on overcoming this challenge through innovative protein engineering strategies. For example, the Chen et al. successfully enhanced proper folding and achieved a 40% increase in soluble expression by fusing UGT76G1 with an N-terminal CysQ solubility tag (134). In another approach, Shu et al. and colleagues used an Smt3 fusion tag along with co-expression of endogenous chaperones, resulting in a remarkably high soluble yield of 1.97 g/L for the Smt3-UGT76G1 fusion protein (135). Beyond improving solubility, engineering efforts have also focused on optimizing catalytic performance. Using structure-guided rational design, the Guo et al. developed a UGT76G1 variant (T284S/M88L/L200A) that enhances substrate-binding conformation; this mutant achieved a 90.5% pilot-scale conversion yield of Reb M (136). Collectively, these advancements emphasize the critical role of protein engineering in enabling the industrial application of UGT76G1.

### 8.4 A multi-enzyme collaborative strategy to optimize Reb M biosynthesis

Relying solely on UGT76G1 for the biosynthesis of Reb M often results in the formation of undesired byproducts due to the enzyme’s inherent catalytic promiscuity. Introducing additional, more specific UGTs provides a promising strategy to streamline the biosynthetic pathway and enhance both yield and purity. For example, the Zhang et al. innovatively employed a rice-derived glycosyltransferase, OsUGT91C1, which demonstrates high regioselectivity for β(1–2) glucosylation. This enzyme efficiently reducing the generation of byproducts with β(1–6) linkages. As a result, facilitating its specific conversion to Reb M (30). Beyond enzyme selection, process engineering also plays a crucial role. Wang et al. developed an advanced co-immobilization system using recombinant OsEUGT11 and SrUGT76G1. This system not only increased the overall conversion efficiency from Reb A to Reb M but also greatly improved the operational stability and recyclability of the enzymes—key advantages for scaling up to cost-effective industrial biomanufacturing (129). Together, these approaches highlight how optimizing pathway specificity and enzyme stability can synergistically overcome the limitations of single-enzyme catalysis.

### 8.5 Industrialization bottlenecks and future research priorities

Despite groundbreaking scientific advances, the transition of Reb M biosynthesis from laboratory success to cost-competitive industrial production faces interconnected challenges that define key research priorities.

Low conversion efficiency and byproduct formation: Inefficient steps, particularly the final glycosylation by UGT76G1, lead to intermediate accumulation and unwanted byproducts, reducing yield and increasing purification complexity (30).

Enzyme stability and cost: Engineered enzymes may degrade under industrial conditions (e.g., heat, shear stress), and their production/purification costs remain high (134).

Product solubility and process scale-up: Reb M’s poor solubility at high concentrations complicates bioconversion and downstream processing. Scaling up introduces challenges in mass transfer, process control, and contamination (134).

Overcoming the existing bottlenecks in the industrial-scale production of Reb M demands a concerted and multidisciplinary strategy that integrates computational biology, protein engineering, and advanced process design. A key direction lies in AI-guided enzyme design, exemplified by the approach of the Zhao et al., where artificial intelligence and molecular dynamics simulations are employed to analyze glycosyltransferase conformational dynamics during catalysis. This enables the precise prediction of mutations that enhance enzyme stability, solubility, and catalytic specificity—particularly toward the desired β(1–3) glycosidic linkage (137). Complementing this, systematic enzyme optimization HO-1 should combine rational design—using crystal structures of key UGTs such as OsUGT91C1 and UGT76G1—with directed evolution to develop ultra-efficient and highly specific enzyme variants. A critical engineering goal is to significantly enhance the thermostability of HO-1, which is essential for maintaining enzymatic activity under industrial process conditions, thereby reducing cooling requirements and operational costs. From a process standpoint, innovative reactor design—including the development of sophisticated cascade reaction systems and advanced co-immobilization of enzymes on durable supports—can significantly increase total turnover numbers and facilitate continuous biomanufacturing, leading to substantial gains in volumetric productivity. Furthermore, to overcome solubility constraints and mitigate product inhibition, the implementation of *in situ* product removal technologies—such as aqueous-organic biphasic systems or selective adsorption techniques—will be crucial for continuously extracting Reb M from the reaction environment. This approach shifts reaction equilibrium forward, maximizes conversion yields, and simplifies downstream purification. Together, these integrated efforts form a comprehensive roadmap toward economically viable and sustainable Reb M production.

## 9 Discussion

Although research has uncovered the diverse pharmacological effects of stevia, particularly its extracts and isolated active components—including antioxidant, anti-inflammatory, anti-hyperglycemic, anti-hyperlipidemic, anti-hypertensive, anti-caries, antimicrobial, anti-cancer, anti-diarrheal properties, as well as liver and kidney protectionred the diverse pto recognize the significant limitations of current evidence, which casts uncertainty on its clinical application. Most conclusions stem from animal or cellular studies that utilize doses far exceeding the ADI for humans, often involving limited sample sizes. The complex chemical profile of stevia extracts or their derived compounds adds another layer of difficulty when it comes to standardization and mechanistic investigations. Therefore, translating these preliminary findings into definitive health benefits for humans requires rigorous future clinical trials. Such studies should involve large cohorts, standardized preparations, and strict adherence to the ADI safety limit of 4 mg per kg body weight, as established by authoritative organizations like JECFA. They must focus on examining the preventive or ameliorative effects of stevia components at doses that reflect real-world consumption, specifically targeting critical endpoints such as diabetes and cardiovascular diseases while elucidating precise dose-response relationships.

From regulatory and consumer perception perspectives, a global scientific consensus has laid a foundation for the use of SGs. Consumers show a clear preference for products with natural ingredients and clean labels. For a natural sweetener to be viable for widespread use and commercial success, it must fulfill several key criteria: ensuring safety, delivering excellent flavor, offering high solubility and stability, and maintaining a reasonable and cost-effective application profile (138). High-purity stevia leaf extract has been approved for use in food and beverages by more than 150 countries and regions (45). Major regulatory bodies worldwide (e.g., EFSA, JECFA) apply a unified ADI safety standard consistently to SGs produced through both traditional extraction methods and novel biomanufacturing techniques, such as enzymatic conversion and microbial fermentation, based on the “common metabolic pathway” principle—provided that the final products are chemically identical (25, 107). This approach has facilitated the commercialization of high-quality components like Reb M. A survey indicates that consumers hold a positive perception of stevia-sweetened products and are willing to accept them and adapt their dietary habits accordingly (139, 140). However, a significant gap persists between the scientific consensus and public understanding. Many consumers remain unaware of stevia-based sweeteners, a knowledge gap particularly pronounced among populations with lower education levels in developing countries (141). Thus, clearer labeling practices from the industry, optimized regulatory requirements, and continued public education in science are vital to bridging this knowledge gap and building evidence-based consumer trust.

Based on this analysis, future development priorities should focus on three interconnected core areas. First, addressing critical research gaps is paramount; this includes conducting large-scale, long-term randomized controlled trials aimed at hard clinical endpoints and investigating the long-term effects of SGs on the human gut microbiome. Second, enhancing technological translation and safety assessment is essential; this involves overcoming production challenges related to Reb M through protein engineering and process optimization, as well as establishing systematic and independent safety profiles for each novel engineered fermentation strain, including assessments of impurity profiles and potential risks. Finally, advancing our understanding of fundamental mechanisms and refining risk assessment is necessary; this encompasses clarifying how SG metabolites interact with key human biological systems, such as renal transporters, and initiating long-term, low-dose exposure studies targeting vulnerable populations like pregnant women and infants. In summary, unlocking the full potential of *Stevia rebaudiana* hinges on the collaborative advancement of clinical medicine, synthetic biology, chemical engineering, and modern toxicology—utilizing robust scientific evidence to overcome application barriers and strengthen safety assurances.

## 10 Conclusion

Translating stevia into safe and effective health applications requires further dose-response characterization, rigorous clinical validation, and cost-effective advances in its biosynthesis.
