# Supplementary material for: The progress on stevia (Stevia rebaudiana Bertoni): chemical composition, pharmacokinetics, pharmacological effects, safety, applications, and biosynthesis
Source: Front Nutr. 2026 Feb 16;13:1728578. doi: 10.3389/fnut.2026.1728578 (PMC12950799; doi:10.3389/fnut.2026.1728578)
Supplement: Supplementary file 1 [file Table_1.docx]

**Supplementary Table S1. Detailed Search Strategy and Initial Hit Counts for the Narrative Review on *Stevia rebaudiana* Bertoni.**

| **Search Query** | **Database** | | | **Number** |
| --- | --- | --- | --- | --- |
|  | **Pubmed** | **Web of Sciences** | **CNKI** |  |
| *Stevia rebaudiana* Bertoni | 366 | 1250 |  | 1616 |
| stevia | 1300 | 3157 |  | 4457 |
| steviol glycosides | 639 | 1130 |  | 1769 |
| stevioside | 732 | 1535 |  | 2267 |
| rebaudioside | 497 | 1113 |  | 1610 |
| 甜叶菊 |  |  | 1278 | 1278 |
| 甜菊糖苷 |  |  | 571 | 571 |
| 瑞鲍迪苷 |  |  | 29 | 29 |
| ("Stevia" OR "steviol glycosides" OR "rebaudioside") AND ("applications") | 99 | 229 |  | 328 |
| ("Stevia" OR "steviol glycosides" OR "rebaudioside") AND ("pharmacokinetics") | 29 | 30 |  | 59 |
| ("Stevia" OR "steviol glycosides" OR "rebaudioside") AND ("safety") | 176 | 212 |  | 388 |
| ("Stevia" OR "steviol glycosides" OR "rebaudioside") AND ("metabolism") | 507 | 48 |  | 555 |
| ("Stevia" OR "steviol glycosides" OR "rebaudioside") AND ("toxicity") | 80 | 136 |  | 216 |
| ("Stevia" OR "steviol glycosides" OR "rebaudioside") AND ("Acceptable Daily Intake") | 39 | 51 |  | 90 |
| ("Stevia" OR "steviol glycosides" OR "rebaudioside") AND ("mutagenicity") | 6 | 15 |  | 21 |
| ("Stevia" OR "steviol glycosides" OR "rebaudioside") AND ("carcinogenicity") | 5 | 16 |  | 21 |
| ("Stevia" OR "steviol glycosides" OR "rebaudioside") AND ("biosynthesis") | 141 | 297 |  | 438 |
| ("Stevia" OR "steviol glycosides" OR "rebaudioside") AND ("glycosyltransferase") | 49 | 81 |  | 130 |
| ("Stevia" OR "steviol glycosides" OR "rebaudioside") AND ("UGT76G1") | 45 | 67 |  | 112 |
| ("Stevia" OR "steviol glycosides" OR "rebaudioside") AND ("sweetener") | 463 | 1125 |  | 1588 |
| ("Stevia" OR "steviol glycosides" OR "rebaudioside") AND ("functional food") | 19 | 42 |  | 61 |
| ("Stevia" OR "steviol glycosides" OR "rebaudioside") AND ("animal feed") | 13 | 7 |  | 20 |
| **Sumary** | **5205** | **10541** | **1878** | **17624** |

Note: Hit counts are prior to removal of duplicates and screening for relevance. CNKI searches were conducted using corresponding Chinese keywords.
